# The Extinction Dynamics of Bacterial Pseudogenes

**DOI:** 10.1371/journal.pgen.1001050

**Published:** 2010-08-05

**Authors:** Chih-Horng Kuo, Howard Ochman

**Affiliations:** Department of Ecology and Evolutionary Biology, University of Arizona, Tucson, Arizona, United States of America; University of Michigan, United States of America

## Abstract

Pseudogenes are usually considered to be completely neutral sequences whose evolution is shaped by random mutations and chance events. It is possible, however, for disrupted genes to generate products that are deleterious due either to the energetic costs of their transcription and translation or to the formation of toxic proteins. We found that after their initial formation, the youngest pseudogenes in *Salmonella* genomes have a very high likelihood of being removed by deletional processes and are eliminated too rapidly to be governed by a strictly neutral model of stochastic loss. Those few highly degraded pseudogenes that have persisted in *Salmonella* genomes correspond to genes with low expression levels and low connectivity in gene networks, such that their inactivation and any initial deleterious effects associated with their inactivation are buffered. Although pseudogenes have long been considered the paradigm of neutral evolution, the distribution of pseudogenes among *Salmonella* strains indicates that removal of many of these apparently functionless regions is attributable to positive selection.

## Author Summary

Pseudogenes have traditionally been viewed as evolving in a strictly neutral manner. In bacteria, however, pseudogenes are deleted rapidly from genomes, suggesting that their presence is somehow deleterious. The distribution of pseudogenes among sequenced strains of *Salmonella* indicates that removal of many of these apparently functionless regions is attributable to their deleterious effects in cell fitness, suggesting that a sizeable fraction of pseudogenes are under selection.

## Introduction

One of the most distinctive features of bacterial genomes is their high coding densities, in which genic regions typically constitute more than 80% of the total genome [1]. This is in sharp contrast to many eukaryotes whose genomes contain vast stretches of non-coding DNA and a multitude of transposable and repetitive elements, with protein-coding regions often accounting for only 1% of the genome [2]. The paucity of non-coding regions in bacterial genomes has lead to the idea that pseudogenes would be exceedingly rare [3]; however, recent large-scale analyses have found that virtually all bacterial genomes contain disrupted and eroded genes that have full-length counterparts in other related genomes [4]–[8]. Pseudogenes are particularly prevalent in those bacterial species that have recently become associated with or dependent upon eukaryotic hosts [9]–[10], and in the most extreme cases, pseudogenes can number in the 1,000 s and occupy over half of the genome [11]–[12].

The pseudogenes in bacterial genomes are continually created from ongoing mutational processes and are subject to degradation, and eventual removal, by the further accumulation of mutations. However, the most surprising aspect of bacterial pseudogenes is that their retention time appears to be extremely short. Even in comparison of very closely bacteria, there are very few pseudogenes that are shared among strains typed to the same bacterial species [6], [7], [13]. This observation indicates that bacterial pseudogenes, although often present in high numbers, are deleted at a relatively rapid rate. This feature is again in sharp contrast to eukaryotes, in which pseudogenes often persist over evolutionary timescales and may be shared by distantly related lineages, such as rodents and primates [14]–[17].

Due to the pervasive mutational bias towards deletions that has been observed across bacterial genomes [18]–[19], the rapidly removal of pseudogenes could be caused by the random fixation of background mutations. Because pseudogenes have long been viewed as “a paradigm of neutral evolution” [20], this is the favored hypothesis. Alternatively, pseudogenes could effect a cost and be eliminated from bacterial genomes by an adaptive process. For example, pseudogenes might be detrimental to the organism through energetic costs incurred by the continued transcription and translation of non-functional genes and/or through the production of proteins that are toxic to cells.

In this study, we examine the formation, loss and phylogenetic distribution of disrupted genes in *Salmonella*. We focus on this bacterial genus because: (1) high-quality genomic sequences of several *Salmonella* strains have been determined, (2) *Salmonella* genomes, like those of most other pathogens, possess considerable numbers of pseudogenes [21]–[22], (3) the population structure of *Salmonella enterica* is essentially clonal [23], allowing the resolution of an unambiguous strain phylogeny, and (4) both experimental [24] and comparative [25]–[27] studies provide evidence of a strong deletional bias in *Salmonella*, such that genes that are not maintained by selection are rapidly inactivated and eliminated by mutational events. And it is against this background that we test the possibility that the removal of bacterial pseudogenes is adaptive.

## Results

### The phylogenetic distribution of pseudogenes

**Figure 1:**
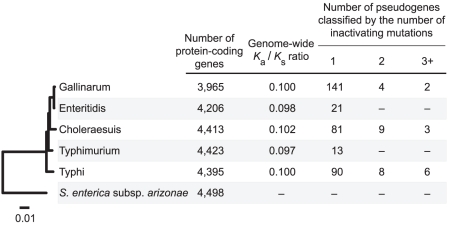
Distribution of pseudogenes among *Salmonella* genomes. The phylogenetic tree was inferred from 2,898 single-copy genes shared by all five *S. enterica* subsp. *enterica* strains and the outgroup *S. enterica* subsp. *arizonae*.

Despite the similarity in overall genome sizes, the number of pseudogenes identified in the five *Salmonella enterica* subsp. *enterica* genomes can vary by an order of magnitude, ranging from 13 in *S. enterica* sv. Typhimurium to 147 in *S. enterica* sv. Gallinarum (Figure 1). The abundance of pseudogenes is not associated with divergence time or phylogenetic affiliations. In fact, the two most closely-related strains, *S. enterica* sv. Gallinarum and *S. enterica* sv. Enteritidis, represent nearly the observed extremes of pseudogene abundance (147 versus 21).

Interestingly, the abundance of pseudogenes in the *Salmonella* genomes is reflected in the genome-wide *K*_a_/*K*_s_ ratio, which can serve as a proxy for measuring the level of genetic drift experienced by a lineage. For example, despite their independent origins, the three strains with the highest numbers of pseudogenes also have the highest genome-wide *K*_a_/*K*_s_ ratios. This observation is consistent with the expectation that deleterious mutations, such as inactivation of functional genes, are more likely to reach fixation in populations under high levels of genetic drift. In addition to higher rates of pseudogene production, high levels of genetic drift may also result in slower rates of pseudogene removal (assuming that the removal of pseudogenes is favored by positive selection). Consistent with this expectation, highly degraded pseudogenes with multiple inactivating mutations were only found in the three strains with high genome-wide *K*_a_/*K*_s_ ratios. But despite the high correlation coefficient between genome-wide *K*_a_/*K*_s_ ratios and the abundance of pseudogenes in these genomes (*r* = 0.74), the correlation does not reach statistical significance (*P* = 0.15), possibly because of the limited number of genomes examined or the use of a distant outgroup limits the resolution in *K*_a_/*K*_s_ ratio estimation (*i.e.*, the difference in high *vs*. low *K*_a_/*K*_s_ groups is underestimated because most substitutions occurred on the branch leading to the outgroup).

Consistent with previous studies in other bacterial genera [6], [7], [13], most pseudogenes in *Salmonella* genomes are strain-specific. Of the 147 pseudogenes in the *S. enterica* sv. Gallinarum genome, only five are shared with its closest relative, *S. enterica* sv. Enteritidis. And of these five, only three share the same inactivating mutations and can be inferred as ancestral. The remaining two shared pseudogenes have different inactivating mutations and were inferred to result from independent events.

### The type and frequency of gene-inactivating mutations

**Figure 2:**
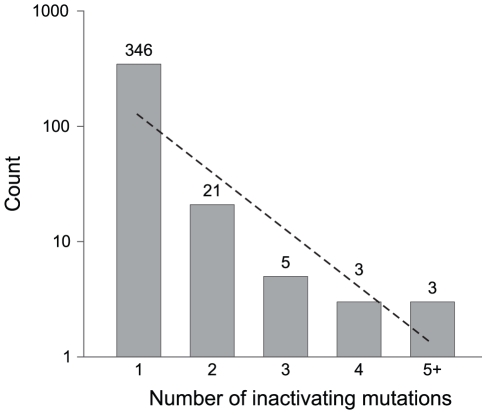
Pseudogenes classified by the number of gene-inactivating mutations. Numbers on top of columns indicate actual counts. The dashed line represents the neutral expectation based on an exponential regression.

To examine mutational processes responsible for creating pseudogenes, we characterized each pseudogene in these *Salmonella* genomes by the number and type of gene-inactivating mutations. The vast majority of *Salmonella* pseudogenes (346/378) have only a single inactivating mutation (Figure 2), and among these, short deletions that removed 20% or less of the original open-reading frame predominate (141/346). We observed two cases of complete removal, including a 2,557-bp deletion containing a 1,224-bp gene and a 441-bp deletion encompassing a 189-bp gene. Because we required a pseudogene to be flanked by two conserved genes for its identification, any pseudogene that was removed by a deletion including a neighboring gene would not be recognized by this synteny-based approach. As expected from the mutational bias towards deletions in bacterial genomes, only a small fraction (17%) of the 346 pseudogenes were produced by an insertion; and with the exception of two cases of transposon insertions, all insertions are <10 bp. All remaining cases are due to point mutations: in 131 cases, there is a premature stop codon that reduced the length of the open reading frame by more than 20% and in two cases, a point mutation altered the start codon (one ATG to ATA, and one ATG to ATT).

We identified 32 pseudogenes with more than one inactivating mutation. There is no significant difference in average gene length between pseudogenes containing multiple inactivating mutations and those with a single inactivating mutation. Detailed information of the 378 curated pseudogenes is presented in Table S1.

### The age distribution of pseudogenes

**Figure 3:**
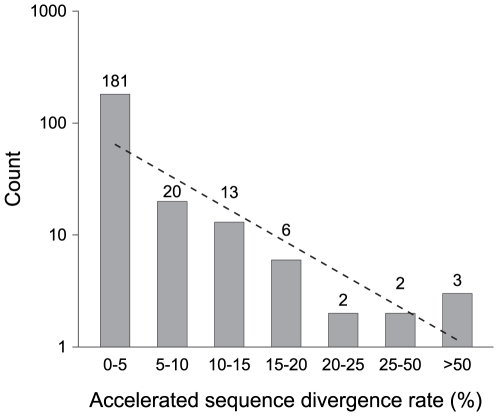
Pseudogenes classified by their acceleration in rates of sequence divergence. Two functional orthologs, one from the outgroup (*S. enterica* subsp. *arizonae*) and one from the nearest ingroup, were used in multiple sequence alignments to calculate the relative sequence divergence rate for each pseudogene. Numbers on top of columns indicate actual counts. The dashed line represents the neutral expectation based on an exponential regression.

Because neutral sequences accrue mutations with time, the relative age of a pseudogene is reflected in its number of accumulated mutations. The preponderance of pseudogenes with a single inactivating mutation indicates that most pseudogenes in these genomes are very young (Figure 2). This is also supported by the fact that most pseudogenes are restricted to individual genomes, as expected if they are newly formed. Further attesting to the recency of most pseudogenes is that there is little or no sign of accelerated sequence divergence relative to their functional orthologs (Figure 3).

Given the strong deletional bias in bacterial genomes, it is possible that the lack of old pseudogenes results from the rapid elimination of non-functional sequences by random fixation of mutations alone. If the jettisoning of pseudogenes is largely governed by a strictly neutral process, we expect that the probability of pseudogene removal to be independent to its age. Under this scenario, the age-class distribution is expected to decrease linearly in a log-normal plot (*e.g.*, Figure 2 and Figure 3). However, there is an overabundance of young pseudogenes relative to this expectation no matter which one of the three methods we used to assign age class (phylogenetic distribution, number of inactivating mutations, and level of accelerated sequence divergence). This indicates that a non-neutral force is operating to remove young pseudogenes such that few remain in the genome long enough to accumulate multiple inactivating mutations or to exhibit accelerated sequence divergence rates (as would be expected for non-functional regions that were released from selective constraints). Consistent with this hypothesis, we detected a strong negative correlation between the loss rate and the age of pseudogenes estimated by the number of inactivating mutations (*r* = −0.99, *P* = 0.013). The scarcity of old pseudogenes is not likely to be an artifact of the methodologies used to identify pseudogenes (or, as noted above, the methods used to assign pseudogene age): our synteny-based approach is capable of detecting highly degraded pseudogenes harboring more than 10 frameshift mutations [19].

### Inference of possible protein–protein interactions

**Figure 4:**
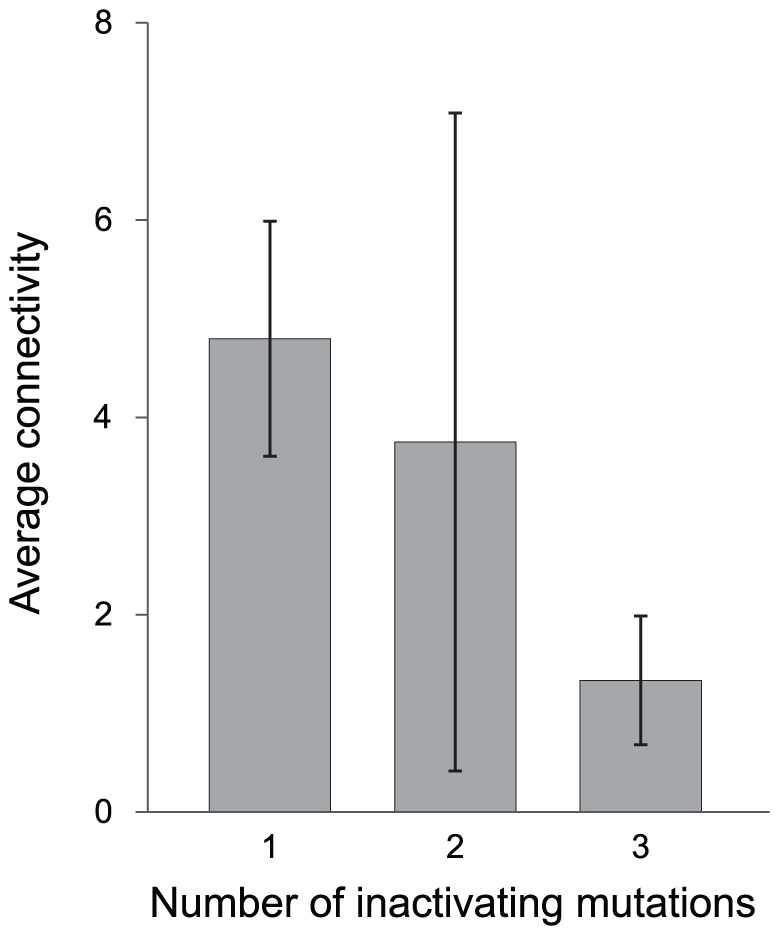
Inferred connectivity of pseudogene homologs in protein–protein interaction networks. The connectivity of each pseudogene was inferred from its functional ortholog in *Escherichia coli* str. K-12 substr. MG1655 (see Methods). Error bars denote 95% confidence intervals. The difference in number of interacting partners between pseudogenes with a single inactivating mutation and those with three inactivating mutations is statistically significant (*P* = 0.003, one-tailed unpaired t-test assuming unequal variance).

Of the 378 identified pseudogenes among *S. enterica* genomes, 120 have functional orthologs in *Escherichia coli str.* K-12 *substr*. MG1655 for which protein-protein interaction data are available. The average numbers of interacting partners in the protein-protein interaction network (*i.e.*, the connectivity) for pseudogenes having different numbers of inactivating mutations revealed that highly degraded pseudogenes (*i.e.*, those with three inactivating mutations) have, on average, significantly fewer interacting partners that do newly formed pseudogenes (*i.e.*, those with a single inactivating mutation) (Figure 4).

## Discussion

If pseudogenes are completely functionless and their eliminations from bacterial genomes were governed by a strictly neutral process, the time since gene inactivation would not influence the probability of pseudogene removal from a genome. However, examination of pseudogene occurrence across multiple *Salmonella* genomes revealed deviations from a model of stochastic loss. Several independent lines of evidence, including the phylogenetic distribution of pseudogenes and the pattern of mutation accumulation, each demonstrated that newly formed pseudogenes were purged from bacterial genomes faster than neutral expectation, suggesting that they confer deleterious effects.

### Non-neutral evolution of bacterial pseudogenes

A possible explanation for this violation of neutrality is that there is selection for minimizing the size of mutational targets [28]; and since bacteria often have larger effective population sizes than do eukaryotes [29], it might be possible for selection to operate on mutations with extremely small effects (*e.g.*, a 1-kb pseudogene accounts for only about 0.02% of a *Salmonella* genome). Unfortunately, this hypothesis cannot explain the observed pattern: If selection against inert DNA were the primary factor causing the removal of pseudogenes, we would expect to find fewer, but not necessarily a higher loss rate, for young pseudogenes. The age-distribution pattern predicted by this mutational-target model would, in fact, be indistinguishable from a strictly neutral model.

To account for differences in loss rate among pseudogenes belonging to various age classes requires methods that can accurately determine the relative ages of the pseudogenes present in this bacterial clade. Because most mutations accumulate as a function of time, one method was to use the level of sequence degradation as an indicator of pseudogene age. Because the youngest pseudogenes, *i.e*., those containing only a single inactivating mutation, have a higher probability of being expressed, their increased loss rate could result from the energetic costs of transcription and translation, which are known to shape the genome organization in prokaryotes [30]–[32] and eukaryotes [33].

Because bacterial cells are haploid, all mutations are effectively dominant since non-functional gene products cannot be masked by the corresponding functional allele as in diploid organisms. In that short indels (*i.e*., less than 10-bp) that cause frameshift and pre-mature stop codons are two of the most common types of mutations observed among bacterial pseudogenes (see Results and [6]–[7]), it is likely that the products from these altered open reading frames are disruptive to normal operation of cellular networks. This ‘toxic protein’ hypothesis is supported by our inference of protein-protein interactions: We find that those few pseudogenes that have persisted in *Salmonella* genomes (*i.e.*, those that have accumulated multiple inactivating mutations) correspond to genes with relatively few interacting partners (Figure 4). The low connectivity of these genes can perhaps serve to minimize the deleterious effects of their inactivation. In contrast, for genes with large numbers of interacting partners, alteration of the open reading frames would potentially impact many protein-protein interactions. As such, mutations that remove such pseudogenes would be highly favorable and quickly reach fixation in the population. The negative correlation observed between loss rate and the age of pseudogenes is consistent with this model.

### Expression and elimination of pseudogenes

The premise that pseudogenes are removed because their encoded products are either energetically costly or toxic relies on the assumption that, after their initial disruption, these sequences are still being transcribed and translated. The majority of pseudogenes that we analyzed are newly arisen (*i.e.*, have a single inactivating mutation in their coding regions), and since the mutational target of the regulatory portion of a gene is much smaller than the coding region, they are unlikely to harbor mutations that affect their expression. This is confirmed by the fact that most of these pseudogenes have nearly 100% sequence identity to their functional orthologs across the entire upstream intergenic region (*i.e.*, from the end of the anchoring gene to the start codon).

As originally observed in *E. coli* [34], [35], and recently shown to occur in other bacteria [36], virtually all (even antisense) sequences in bacterial genomes are transcribed. Direct evidence of pseudogene expression is available for several strains of *Salmonella*. In a global analysis of Typhimurium gene expression using microarrays, Hautefort *et al*. [37] reported values for the relative expression of about 4,000 genes during host-cell infection. Some pseudogenes were up-regulated (*e.g., putA, rffH*), and others were down-regulated (*e.g., dgoA*), more than two-fold under the experimental conditions. An RNA-seq analysis of Typhi found that many pseudogenes were transcribed, albeit at highly reduced levels [38]. In this analysis, nine Typhi pseudogenes– both young and old – were still expressed high levels, but the overall reduction in pseudogene expression was taken to indicate that the majority of pseudogenes were no longer active [38], possibly to ameliorate the deleterious effects that we detected.

To determine if reduced expression fosters the maintenance of pseudogenes in bacterial genomes, we examined the codon adaptation index (CAI, which is an indicator of overall expression levels over evolutionary timescales) of genes in the difference age classes. Paralleling the effect shown in Figure 4, the average CAI is significantly lower in older pseudogenes (average CAI  = 0.29 for age class 3 vs. 0.36 for age class 1; *p* = 0.003, one-tailed unpaired t-test assuming unequal variance). These results are consistent with our expectation that selection acts to remove more highly expressed (and connected) genes once they become pseudogenized.

### Ridding bacterial genomes of pseudogenes

Mutations in bacterial genomes are known to be highly biased toward deletions [18], [19]. Therefore, it is not surprising to find that accumulation of deletions is the primary force responsible for the erosion of bacterial pseudogenes. However, only a small fraction of pseudogenes detected in *Salmonella* genomes were found to have lost more than 20% of their original length, despite the high sensitivity of our synteny-based method for pseudogene detection. Given the high incidence of kilobase-sized (and larger) deletions observed during *Salmonella* experimental evolution [24], the main mechanism for the complete removal of pseudogenes is likely to be large deletions, most of which are large enough to remove neighboring genes and therefore cannot be detected using a local-synteny based approach.

### Conclusion

Our systematic characterization of multiple *Salmonella* genomes indicates that the evolution of bacterial pseudogenes is not strictly neutral such that newly formed pseudogenes have a higher likelihood of being removed. This deviation from the generally accepted view that pseudogenes represent completely neutral regions [20] is likely due to the fact that bacteria have haploid genomes and generally large effective population sizes, therefore increasing the exposure of mutations to selective forces. If pseudogenes are deleterious due either to the energetic costs of transcription and translation or to the dominant-negative effects of anomalous proteins, the high efficacy of selection in bacterial genomes is likely to have a role in their removal. This is consisitent with our finding that those *Salmonella* genomes with the lowest genome-wide *K*_a_/*K*_s_ ratios denoting a relatively high efficacy of selection harbor the lowest numbers of pseudogenes.

Because all bacterial groups, as well as those Archaea examined, display a mutational pattern that is biased towards deletions [18], [19], [33] and their haploid genomes would be more susceptible to dominant-negative effects that pseudogenes might impart, it is likely that the process of adaptive removal of pseudogenes is pervasive among prokaryotes. And given the evidence for selection on intron size in some eukaryotic genomes, presumably due to the energetic cost of transcription [39], these effects need not be restricted to those cellular organisms with haploid genomes, and pseudogene degradation and removal may be found to be operating under similar principles in representatives from all domains of life.

## Materials and Methods

### Data source

We obtained the complete genome sequences of six *Salmonella enterica* strains from NCBI GenBank [40], including *S. enterica* subsp. *enterica* serovar Enteritidis str. P125109 (NC_011294), *S. enterica* subsp. *enterica* serovar Gallinarum str. 287/91 (NC_011274), *S. enterica* subsp. *enterica* serovar Choleraesuis str. SC-B67 (NC_006905), *S. enterica* subsp. *enterica* serovar Typhimurium str. LT2 (NC_003197), *S. enterica* subsp. *enterica* serovar Typhi str. CT18 (NC_003198), and *S. enterica* subsp. *arizonae* serovar 62:z4,z23:– (NC_010067) as the outgroup. This set of genome sequences were selected because: (1) the low level of divergence allows for reliable sequence alignment and thus confident inference of gene inactivation events, (2) the phylogenetic relationship among the six strains allows for straightforward assignment of age-class for pseudogenes base on their phylogenetic distribution pattern, and (3) the sequencing was performed by high-coverage whole-genome shotgun sequencing with the Sanger method, which provides high accuracy in homopolymer regions. The last point was of particular importance because our preliminary analysis suggests that sequencing errors in homopolymer regions are a major factor that contributes to erroneous pseudogene annotations in several other *S. enterica* genome sequences. The difficulties involved in distinguishing true frameshift mutations from sequencing errors prohibit a more comprehensive taxon sampling.

### Ortholog identification

To identify orthologous gene shared among the six *S. enterica* genomes, we performed all-against-all NCBI-BLASTN [41] searches with an e-value cutoff of 1×10^−15^ for all annotated protein-coding genes. A set of custom Perl scripts written with Bioperl modules [42] were used for data parsing and processing. The BLASTN results were supplied as the input for OrthoMCL [43] to perform ortholog clustering. The algorithm is largely based on the popular criterion of reciprocal best hits between genomes and has been shown to perform well by a benchmarking study [44].

### Phylogenetic inference

To infer the phylogenetic relationship among the six *S. enterica* strains, we aligned the nucleotide sequences of the 2,898 single-copy genes shared by all six strains using MUSCLE [45] with the default parameters. We used TREE-PUZZLE [46] to infer the distance matrix and the phylogenetic tree based on a concatenated alignment with 2,772,598 sites. The changes from default setting in TREE-PUZZLE include: (1) use exact parameter estimates, (2) estimate the nucleotide frequencies and transition/transversion ratio from the data set, (3) use a mixed model with one invariable and eight Gamma rates for rate heterogeneity, and (4) estimate the fraction of invariable sites and the Gamma distribution parameter from the data set.

### Genome-wide substitution rate estimates

We calculated the genome-wide *K*_a_/*K*_s_ ratio for each of the five ingroup strains to estimate the level of genetic drift experience by the lineage. This ratio is a good approximation for the level of genetic drift because it measures the efficacy of purifying selection in protein-coding region; an elevated level of genetic drift can result in increased incidence of slightly deleterious amino acid replacement, and thus, an increase in genome-wide *K*_a_/*K*_s_ ratio. Although positive selection favoring certain amino acid changes can also increase *K*_a_, such scenario is expected to be limited to particular genes and sites rather than driving changes throughout the entire genome [1], [47].

For each of the 2,898 single-copy genes shared by all six strains, we performed multiple sequence alignment at amino acid level using MUSCLE [44] with the default parameters. The resulting protein alignments were converted into codon-based nucleotide alignment using PAL2NAL [48]. To account for possible base composition and codon usage bias in any of the genes examined, we applied the YN00 method [49] implemented in the PAML package [50] to estimate the substitution rates. For each of the five ingroup strains, we calculated *K*_a_ and *K*_s_ using the outgroup *S. enterica* subsp. *arizonae* as the reference. To avoid potential bias in *K*_a_/*K*_s_ ratio estimation due to non-sufficient sequence divergence or saturation, we removed genes that have an estimated *K*_s_ of less than 0.1 or greater than 1.5 in any of the five pair-wise comparisons. The average *K*_a_/*K*_s_ ratio calculated from the 2,290 remaining genes was used to represent the genome-wide estimate for each of the five ingroup strains.

### Pseudogene identification and curation

We utilized a synteny-based approach similar to that described previously [19] for pseudogene identification. Although this approach may underestimate the total number of pseudogenes in a genome due to the exclusion of pseudogenes that lack positional homologs in other closely related genomes (which may have originated from horizontal transfer), the stringent requirement allows for confident inference of the gene inactivation events.

To identify pseudogenes with positional homologs, each of the five *S. enterica* subsp. *enterica* strains was used as the query against every other genome. The outgroup *S. enterica* subsp. *arizonae* was not considered as a query because the ancestral state of any pseudogene identified in this genome cannot be established with our taxon sampling. For each pair of query and subject, we utilized single-copy genes shared by the two genomes as anchors to systematically examine the intergenic regions in the query genome. An intergenic region is flagged as containing a putative pseudogene if an annotated protein-coding gene was found in the syntenic region of other genomes.

For each candidate region, we aligned the query genome to the subject genome using MUSCLE [45] with the default parameters. The two anchoring genes were included to improve the quality of alignment and to allow for examination of the entire intergenic region of the query genome. Possible gene-inactivating mutations, including insertions, deletions, pre-mature stop codons, and/or point mutations in the start codon were inferred based on the annotated gene in the subject genome. The results were manually inspected for the consistency regarding gene synteny and the identified mutations across different reference genomes.

To ensure a high level of confidence when inferring gene-inactivating mutations, we required at least two positional homologs to establish the ancestral state of a pseudogene. During our curation process, the following types of false-positives were removed before the final analysis: (1) the entire open reading frame is intact in the query genome but not annotated as a gene, (2) the putative pseudogene may be explained as an annotation artifact (*e.g.*, the region was annotated as a part of either anchoring genes in the query genome), (3) pre-mature stop codons are the only type of mutations and the protein lengths were reduced by less than 20%, (4) the reference gene is a transposase from a insertion sequence element or of viral-origin (*i.e.*, likely a gene gain in the subject genome instead of a gene loss in the query genome), (5) the phylogenetic distribution of the reference gene suggests that a single gene gain event (*e.g.*, horizontal gene transfer) is the most likely explanation for the absence of corresponding gene in the query genome, and (6) the reference gene is a poorly conserved hypothetical protein and is shorter than 300 bp.

In the rare cases where the identified mutations exhibit inconsistency across different reference genomes or indicate extensive sequence divergence, we extracted the syntenic region from all genomes to perform multiple sequence alignment and deduced the inactivation events based on the most parsimonious scenario. One special case involved a 873-bp inversion within the srfB pseudogene in the Gallinarum genome; we manually corrected the inversion before the multiple sequence alignment to infer other possible inactivation events. Furthermore, the exact boundaries of all indel events that affect the 5′- or 3′-end of a pseudogene were manually verified.

To classify the curated pseudogenes into different age classes, we examined their phylogenetic distribution pattern to characterize the likely time point of gene inactivation events on the phylogeny. Because 302 out of the 378 curated pseudogenes are specific to one genome, we used two additional methods for age class assignments. In the first method, we categorized the pseudogenes based on the number of gene-inactivating mutations that have been accumulated. In the second method, we utilized an ortholog in the outgroup to quantify the level of accelerated sequence divergence in the pseudogene relative to its functional ortholog in another genome. The nucleotide sequence alignments were inferred using MUSCLE [45] with the default parameters and subsequently used as the input for TREE-PUZZLE [46] to calculate distance matrices. Due to the lack of appropriate orthologs in the outgroup, only 227 out of the 378 curated pseudogenes can be classified using this method.

### Protein–protein interaction inference

To infer the potential role of a *Salmonella* pseudogene in cellular fitness, we identified the orthologous gene in *Escherichia coli* str. K-12 substr. MG1655 (NC_000913), a related enteric strain on which extensive experimental and functional assays have been conducted [51], [52]. For ortholog identification, we used the full-length gene from the closest reference genome as the query to perform NCBI-BLASTP [40] searches. To qualify as an ortholog, we required the BLASTP hit to satisfy all of the following conditions: (1) is the best hit among all of the protein-coding genes in the *E. coli* MG1655 genome, (2) has an BLASTP e-value of less than 1×10^−15^, (3) the difference in gene length is no more than 20% of the shorter sequence, (4) the high scoring pairs (HSPs) account for at least 80% of the shorter gene, and (5) the fraction of conserved amino acid sites is at least 60% within HSPs. For pseudogenes that had a corresponding ortholog in the *E. coli* MG1655 genome, we extracted the protein-protein interaction information from the high quality combined dataset available from an integrated protein interaction database [53], [54] to infer numbers of interacting partners.

## Supporting Information

Table S1 List of pseudogenes detected and analysed.
(0.25 MB PDF)
