# Supplementary material for: The Extinction Dynamics of Bacterial Pseudogenes
Source: PLoS Genet. 2010 Aug 5;6(8):e1001050. doi: 10.1371/journal.pgen.1001050 (PMC2916853; doi:10.1371/journal.pgen.1001050)
Supplement: Table S1 — List of pseudogenes detected and analysed. (0.25 MB PDF) [file pgen.1001050.s001.pdf]

ID	Query	Subject	Anchor	Query_start	Query_end	Query_strand	Subject_protein	Subject_name
1	Choleraesuis	Typhimurium	76_77	25804	28425	1	NP_459028	bcfC
2	Choleraesuis	Typhimurium	2980_88	44706	46078	-1	NP_459047	NA
3	Choleraesuis	Enteritidis	239_240	277682	278379	1	YP_002242410	NA
4	Choleraesuis	Typhimurium	3017_3018	423519	424278	-1	NP_459331	stbE
5	Choleraesuis	Typhimurium	3837_3838	438433	441600	-1	NP_459346	NA
6	Choleraesuis	Typhimurium	3528_3838	443100	445390	1	NP_459348	NA
7	Choleraesuis	Typhimurium	361_362	578123	578776	1	NP_459472	acrR
8	Choleraesuis	Typhimurium	3050_375	601322	602148	-1	NP_459492	NA
9	Choleraesuis	Typhimurium	3053_3054	621824	623613	1	NP_459512	gcl
10	Choleraesuis	Typhimurium	3070_3071	657923	658235	-1	NP_459546	NA
11	Choleraesuis	Typhimurium	439_440	720572	721811	1	NP_459607	ybdR
12	Choleraesuis	Typhimurium	440_441	722539	723346	-1	NP_459609	rna
13	Choleraesuis	Typhimurium	449_450	734704	735276	1	NP_459620	pagP
14	Choleraesuis	Typhimurium	3846_3847	763583	765046	1	NP_459650	ybeV
15	Choleraesuis	Typhimurium	3081_488	794618	795757	-1	NP_459675	citB
16	Choleraesuis	Typhimurium	3850_4101	855989	856973	-1	NP_459744	NA
17	Choleraesuis	Typhimurium	575_576	920070	920997	1	NP_459803	ybiN
18	Choleraesuis	Typhimurium	608_609	973528	974250	1	NP_459851	mdaA
19	Choleraesuis	Typhimurium	674_675	1065471	1069939	1	NP_459969	mukB
20	Choleraesuis	Typhimurium	686_687	1132619	1134526	1	NP_460036	uup
21	Choleraesuis	Typhimurium	734_736	1195741	1197236	-1	NP_460100	NA
22	Choleraesuis	Typhimurium	3120_737	1200940	1203558	1	NP_460104	NA
23	Choleraesuis	Typhimurium	3126_765	1233828	1234752	1	NP_460140	mviM
24	Choleraesuis	Typhimurium	1111_3553	1448164	1448164	-1	NP_460298	rfc
25	Choleraesuis	Typhimurium	1010_1011	1573972	1575012	1	NP_460425	ydgJ
26	Choleraesuis	Typhimurium	993_994	1605256	1606509	-1	NP_460450	NA
27	Choleraesuis	Typhimurium	970_971	1638356	1639228	-1	NP_460483	yneJ
28	Choleraesuis	Typhimurium	3561_3562	1664264	1667311	-1	NP_460529	fdnG
29	Choleraesuis	Typhimurium	3165_3166	1674507	1678247	1	NP_460536	narZ
30	Choleraesuis	Typhimurium	3159_951	1689239	1689904	-1	NP_460547	yncC

ID	Query	Subject	Anchor	Query_start	Query_end	Query_strand	Subject_protein	Subject_name
31	Choleraesuis	Typhimurium	934_935	1713221	1714202	1	NP_460569	ydcK
32	Choleraesuis	Typhimurium	3566_932	1724859	1726367	-1	NP_460582	NA
33	Choleraesuis	Enteritidis	3699_907	1772857	1773414	-1	YP_002243469	NA
34	Choleraesuis	Typhimurium	3138_907	1774364	1776309	-1	NP_460627	NA
35	Choleraesuis	Typhimurium	3135_904	1784103	1785033	1	NP_460636	NA
36	Choleraesuis	Typhimurium	1192_3575	1901114	1902908	1	NP_460743	NA
37	Choleraesuis	Typhimurium	1277_1278	2023606	2024351	-1	NP_460864	cutC
38	Choleraesuis	Typhimurium	1339_3224	2093643	2094554	-1	NP_460940	yedI
39	Choleraesuis	Enteritidis	1434_1435	2275160	2275688	1	YP_002244242	NA
40	Choleraesuis	Typhimurium	1455_1456	2307478	2308997	-1	NP_461134	mglA
41	Choleraesuis	Typhimurium	1516_1517	2416057	2416703	-1	NP_461236	yfaZ
42	Choleraesuis	Typhimurium	1642_1643	2579763	2580257	-1	NP_461390	eutK
43	Choleraesuis	Typhimurium	1643_1644	2580939	2581834	-1	NP_461392	eutC
44	Choleraesuis	Enteritidis	1661_3287	2605652	2607352	1	YP_002244542	narQ
45	Choleraesuis	Enteritidis	1686_4049	2648332	2654389	-1	YP_002244574	shdA
46	Choleraesuis	Typhimurium	1703_1704	2698805	2700654	-1	NP_461474	hscA
47	Choleraesuis	Typhimurium	1766_1767	2830733	2831953	1	NP_461602	yfiN
48	Choleraesuis	Typhimurium	3328_3329	3071607	3072947	-1	NP_461882	ygcY
49	Choleraesuis	Gallinarum	2011_3908	3198821	3201112	-1	YP_002227804	NA
50	Choleraesuis	Typhimurium	3910_3911	3203881	3205353	-1	NP_461999	NA
51	Choleraesuis	Typhimurium	2121_2122	3360561	3361547	1	NP_462147	yqjG
52	Choleraesuis	Typhimurium	2159_2160	3418476	3419325	-1	NP_462206	folP
53	Choleraesuis	Typhimurium	3615_3616	3512661	3514760	1	NP_462298	NA
54	Choleraesuis	Typhimurium	2705_3475	3539817	3540175	-1	NP_462322	rpsM
55	Choleraesuis	Enteritidis	3932_4188	3648575	3650290	1	YP_002245421	NA
56	Choleraesuis	Typhimurium	2599_2600	3685736	3686845	-1	NP_462465	livK
57	Choleraesuis	Typhimurium	3437_3438	3806495	3807190	1	NP_462577	sgbE
58	Choleraesuis	Typhimurium	2540_3437	3808430	3810385	1	NP_462579	NA
59	Choleraesuis	Typhimurium	2525_2526	3835228	3836538	1	NP_462598	NA
60	Choleraesuis	Typhimurium	3424_3425	3907046	3908094	-1	NP_462667	NA

ID	Query	Subject	Anchor	Query_start	Query_end	Query_strand	Subject_protein	Subject_name
61	Choleraesuis	Typhimurium	3419_3948	3915829	3917021	-1	NP_462675	nepI
62	Choleraesuis	Typhimurium	2475_2476	3927381	3928709	-1	NP_462687	uhpC
63	Choleraesuis	Typhimurium	3413_3630	3964339	3965379	-1	NP_462725	torT
64	Choleraesuis	Typhimurium	2446_2447	3985158	3985414	1	NP_462741	NA
65	Choleraesuis	Typhimurium	2443_2444	3989908	3990839	1	NP_462747	yidZ
66	Choleraesuis	Enteritidis	2304_3631	4044104	4045749	1	YP_002245765	ilvG
67	Choleraesuis	Typhimurium	2256_2257	4106603	4107945	1	NP_462851	NA
68	Choleraesuis	Typhimurium	2238_2239	4127905	4129356	1	NP_462871	trkH
69	Choleraesuis	Typhimurium	2405_2406	4138913	4139820	-1	NP_462878	yihG
70	Choleraesuis	Typhimurium	2395_3397	4165682	4166751	-1	NP_462901	yihS
71	Choleraesuis	Typhimurium	2384_2385	4177803	4180853	-1	NP_462917	fdoG
72	Choleraesuis	Typhimurium	3384_3385	4216542	4218076	1	NP_462955	ego
73	Choleraesuis	Typhimurium	2346_2347	4251152	4252707	1	NP_462985	NA
74	Choleraesuis	Typhimurium	3961_3962	4263182	4265478	1	NP_462994	pflD
75	Choleraesuis	Typhimurium	3964_3966	4308623	4308967	-1	NP_463025	NA
76	Choleraesuis	Typhimurium	2322_3966	4310509	4310509	-1	NP_463027	NA
77	Choleraesuis	Typhimurium	2789_3483	4459894	4462041	-1	NP_463150	fdhF
78	Choleraesuis	Typhimurium	2803_3484	4483128	4483394	-1	NP_463167	NA
79	Choleraesuis	Typhimurium	2850_3972	4549576	4550279	1	NP_463232	yjfJ
80	Choleraesuis	Typhimurium	2871_2872	4575701	4576548	-1	NP_463262	ytfG
81	Choleraesuis	Typhimurium	2882_3499	4590687	4591849	-1	NP_463274	NA
82	Choleraesuis	Typhimurium	2972_73	17869	19967	1	NP_459023	NA
83	Choleraesuis	Typhimurium	3077_3078	754849	756018	1	NP_459642	NA
84	Choleraesuis	Typhimurium	510_511	826398	826784	1	NP_459716	NA
85	Choleraesuis	Typhimurium	3852_564	904183	904743	1	NP_459788	NA
86	Choleraesuis	Typhimurium	727_728	1179980	1181866	1	NP_460087	scsB
87	Choleraesuis	Typhimurium	745_746	1213098	1213605	1	NP_460118	NA
88	Choleraesuis	Typhimurium	3335_3336	3143077	3143944	1	NP_461941	yohM
89	Choleraesuis	Typhimurium	2435_3409	3999872	4001223	-1	NP_462757	NA
90	Choleraesuis	Typhimurium	2432_2433	4007801	4008497	-1	NP_462762	NA

ID	Query	Subject	Anchor	Query_start	Query_end	Query_strand	Subject_protein	Subject_name
91	Choleraesuis	Typhimurium	1421_1422	2234198	2235607	1	NP_461074	yegB
92	Choleraesuis	Enteritidis	2896_2897	4616943	4616969	-1	YP_002246236	NA
93	Choleraesuis	Enteritidis	478_479	779770	779835	-1	YP_002242788	NA
94	Enteritidis	Gallinarum	93_94	58700	59903	1	YP_002225194	NA
95	Enteritidis	Gallinarum	158_2998	161662	162650	-1	YP_002225278	NA
96	Enteritidis	Gallinarum	328_329	471148	472641	1	YP_002225550	NA
97	Enteritidis	Gallinarum	3847_469	696145	697383	-1	YP_002225747	NA
98	Enteritidis	Typhimurium	608_609	904673	905395	1	NP_459851	mdaA
99	Enteritidis	Gallinarum	729_730	1081550	1082791	1	YP_002226071	agp
100	Enteritidis	Gallinarum	3142_930	1500659	1502208	-1	YP_002226480	NA
101	Enteritidis	Gallinarum	1426_3242	2237725	2238625	1	YP_002227080	yegS
102	Enteritidis	Gallinarum	3263_3264	2390911	2392200	-1	YP_002227207	yfaV
103	Enteritidis	Choleraesuis	3289_4376	2635382	2642689	-1	YP_217498	ratB
104	Enteritidis	Choleraesuis	1687_3290	2649589	2651633	-1	YP_217501	sinH
105	Enteritidis	Gallinarum	1833_1834	2875524	2876334	-1	YP_002227589	NA
106	Enteritidis	Gallinarum	2011_3908	3128638	3129591	-1	YP_002227804	NA
107	Enteritidis	Choleraesuis	2036_3913	3159415	3160670	1	YP_218041	nupG
108	Enteritidis	Gallinarum	2411_2412	3967865	3969288	-1	YP_002228333	yieO
109	Enteritidis	Choleraesuis	2287_2288	4003169	4003393	1	YP_218815	rffH
110	Enteritidis	Gallinarum	2397_2398	4094563	4095983	-1	YP_002228202	yihO
111	Enteritidis	Choleraesuis	2384_3395	4117899	4118919	-1	YP_218915	NA
112	Enteritidis	Gallinarum	3477_3479	4285789	4286493	-1	YP_002228783	pepE
113	Enteritidis	Choleraesuis	2804_3634	4409137	4411566	1	YP_219170	dmsA
114	Enteritidis	Gallinarum	2907_2908	4549488	4549977	-1	YP_002229005	ytgA
115	Gallinarum	Enteritidis	2971_67	9982	10695	-1	YP_002242176	htgA
116	Gallinarum	Choleraesuis	3653_74	19947	22943	1	YP_215005	hmt
117	Gallinarum	Enteritidis	2980_88	50336	51708	-1	YP_002242209	NA
118	Gallinarum	Enteritidis	98_99	82263	83816	-1	YP_002242236	caiC
119	Gallinarum	Enteritidis	182_183	200145	202535	-1	YP_002242336	gcd
120	Gallinarum	Enteritidis	3821_3822	207007	209553	-1	YP_002242342	stiC

ID	Query	Subject	Anchor	Query_start	Query_end	Query_strand	Subject_protein	Subject_name
121	Gallinarum	Enteritidis	187_3823	211220	211649	1	YP_002242345	yadI
122	Gallinarum	Enteritidis	3827_3828	237175	237651	1	YP_002242366	stfF
123	Gallinarum	Typhimurium	202_3828	238181	239023	1	NP_459206	NA
124	Gallinarum	Enteritidis	3011_3012	341760	344241	1	YP_002242437	safC
125	Gallinarum	Enteritidis	265_266	363149	364201	-1	YP_002242456	phoE
126	Gallinarum	Enteritidis	3018_3019	379024	381585	-1	YP_002242471	stbC
127	Gallinarum	Enteritidis	269_3529	401318	403271	1	YP_002242490	mod
128	Gallinarum	Enteritidis	291_292	438264	438461	1	YP_002242520	yaiI
129	Gallinarum	Enteritidis	343_344	509002	509398	1	YP_002242585	ybaW
130	Gallinarum	Enteritidis	348_349	514600	516372	1	YP_002242591	mdlA
131	Gallinarum	Enteritidis	380_381	561942	562796	-1	YP_002242634	ybbN
132	Gallinarum	Enteritidis	3839_387	572777	573702	-1	YP_002242644	ybbS
133	Gallinarum	Enteritidis	3062_3063	589270	590934	1	YP_002242658	fdrA
134	Gallinarum	Enteritidis	463_464	696094	697188	1	YP_002242761	cobD
135	Gallinarum	Enteritidis	3078_468	704861	706789	1	YP_002242769	NA
136	Gallinarum	Enteritidis	3539_573	864166	866310	1	YP_002242909	dinG
137	Gallinarum	Enteritidis	578_579	873272	874017	-1	YP_002242917	glnH
138	Gallinarum	Enteritidis	615_616	929909	931036	1	YP_002242966	rumB
139	Gallinarum	Enteritidis	633_634	952693	953807	1	YP_002242987	ybjY
140	Gallinarum	Enteritidis	3114_732	1093535	1093928	1	YP_002243114	ycdC
141	Gallinarum	Enteritidis	736_737	1106244	1107397	1	YP_002243122	NA
142	Gallinarum	Enteritidis	1340_3885	1158564	1159479	-1	YP_002243149	yedA
143	Gallinarum	Enteritidis	1301_3221	1198824	1199149	1	YP_002243193	NA
144	Gallinarum	Enteritidis	1285_1286	1215330	1216989	1	YP_002243211	cheM
145	Gallinarum	Enteritidis	1235_1236	1339956	1341207	-1	YP_002243302	NA
146	Gallinarum	Enteritidis	1227_1228	1348110	1348568	-1	YP_002243312	NA
147	Gallinarum	Enteritidis	1194_3204	1389529	1390377	-1	YP_002243352	hyaF
148	Gallinarum	Enteritidis	885_886	1486356	1487493	-1	YP_002243436	yciW
149	Gallinarum	Enteritidis	886_887	1489086	1490458	1	YP_002243438	NA
150	Gallinarum	Enteritidis	4119_919	1534239	1537764	-1	YP_002243503	nifJ

ID	Query	Subject	Anchor	Query_start	Query_end	Query_strand	Subject_protein	Subject_name
151	Gallinarum	Enteritidis	941_942	1591492	1592441	-1	YP_002243555	sifB
152	Gallinarum	Enteritidis	3168_961	1643668	1646197	1	YP_002243597	NA
153	Gallinarum	Enteritidis	3171_969	1665715	1666663	-1	YP_002243623	NA
154	Gallinarum	Enteritidis	982_983	1684892	1686271	-1	YP_002243643	NA
155	Gallinarum	Enteritidis	3555_40	1691423	1693611	1	YP_002243651	dmsA1
156	Gallinarum	Enteritidis	996_997	1706609	1707862	1	YP_002243664	ynfM
157	Gallinarum	Enteritidis	1030_1031	1753088	1753984	-1	YP_002243708	ydhF
158	Gallinarum	Enteritidis	3882_3883	1804227	1804964	1	YP_002243764	orf245
159	Gallinarum	Enteritidis	1084_1085	1810034	1811035	-1	YP_002243770	ynhG
160	Gallinarum	Enteritidis	1099_1100	1844154	1845588	-1	YP_002243799	ydiU
161	Gallinarum	Enteritidis	1133_3191	1884703	1885164	-1	YP_002243838	astA
162	Gallinarum	Enteritidis	1136_1137	1888596	1889939	1	YP_002243843	gdhA
163	Gallinarum	Enteritidis	1142_4175	1897563	1898920	-1	YP_002243850	NA
164	Gallinarum	Enteritidis	4178_4179	1902747	1903791	-1	YP_002243855	NA
165	Gallinarum	Enteritidis	1164_1165	1926350	1927482	1	YP_002243888	NA
166	Gallinarum	Enteritidis	1176_1177	1940414	1940615	1	YP_002243902	NA
167	Gallinarum	Enteritidis	801_802	1974994	1975510	-1	YP_002243932	ycfJ
168	Gallinarum	Enteritidis	776_777	2004307	2005968	-1	YP_002243960	flgK
169	Gallinarum	Enteritidis	775_776	2006983	2008080	-1	YP_002243962	flgI
170	Gallinarum	Enteritidis	3127_764	2017663	2018309	-1	YP_002243975	yceH
171	Gallinarum	Enteritidis	1352_1353	2095736	2096550	-1	YP_002244106	cbiO
172	Gallinarum	Enteritidis	1362_1363	2108928	2109822	-1	YP_002244122	pocR
173	Gallinarum	Enteritidis	1368_3231	2115359	2117199	1	YP_002244129	pduG
174	Gallinarum	Enteritidis	1372_3585	2119728	2120701	1	YP_002244136	pduO
175	Gallinarum	Enteritidis	1421_1422	2217339	2218750	1	YP_002244216	yegB
176	Gallinarum	Enteritidis	4157_4158	2238366	2240760	-1	YP_002244235	pegC
177	Gallinarum	Enteritidis	1434_1435	2247172	2247700	1	YP_002244242	NA
178	Gallinarum	Enteritidis	1446_1447	2262530	2263292	-1	YP_002244257	yohF
179	Gallinarum	Enteritidis	1449_1450	2271411	2272189	1	YP_002244265	NA
180	Gallinarum	Enteritidis	1455_1456	2279354	2280872	-1	YP_002244274	mglA

ID	Query	Subject	Anchor	Query_start	Query_end	Query_strand	Subject_protein	Subject_name
181	Gallinarum	Enteritidis	1475_1476	2308946	2310502	1	YP_002244300	rtn
182	Gallinarum	Enteritidis	1516_1517	2386687	2387335	-1	YP_002244363	yfaZ
183	Gallinarum	Enteritidis	1572_1573	2454687	2456108	-1	YP_002244428	NA
184	Gallinarum	Enteritidis	1573_1574	2457807	2459233	1	YP_002244430	NA
185	Gallinarum	Enteritidis	1615_1616	2516517	2518705	-1	YP_002244480	yfeA
186	Gallinarum	Enteritidis	1637_1638	2548646	2549221	-1	YP_002244511	NA
187	Gallinarum	Enteritidis	1686_4049	2619296	2625359	-1	YP_002244574	shdA
188	Gallinarum	Choleraesuis	3289_4376	2626053	2633355	-1	YP_217498	ratB
189	Gallinarum	Choleraesuis	1687_3290	2640255	2642448	-1	YP_217501	sinH
190	Gallinarum	Enteritidis	1711_3295	2678021	2679065	1	YP_002244607	asrA
191	Gallinarum	Enteritidis	1712_1713	2680912	2681897	-1	YP_002244610	NA
192	Gallinarum	Enteritidis	1781_1782	2783490	2785669	1	YP_002244689	NA
193	Gallinarum	Enteritidis	1784_1785	2797121	2798366	1	YP_002244696	iroD
194	Gallinarum	Enteritidis	1786_1787	2802581	2803633	-1	YP_002244699	NA
195	Gallinarum	Enteritidis	1788_1789	2806993	2808005	-1	YP_002244702	nxiA
196	Gallinarum	Enteritidis	1794_1795	2814624	2815885	1	YP_002244709	ygaF
197	Gallinarum	Enteritidis	1801_1802	2822994	2823395	-1	YP_002244718	stpA
198	Gallinarum	Enteritidis	1812_3313	2838732	2840269	1	YP_002244735	emrB
199	Gallinarum	Enteritidis	1908_4134	2958212	2960875	-1	YP_002244855	ygcB
200	Gallinarum	Enteritidis	1967_1968	3056669	3057430	-1	YP_002244931	kduD
201	Gallinarum	Enteritidis	2029_2030	3129687	3130694	-1	YP_002245017	yggM
202	Gallinarum	Choleraesuis	2037_3338	3138119	3140250	-1	YP_218042	speC
203	Gallinarum	Enteritidis	2061_2062	3182385	3182908	-1	YP_002245064	NA
204	Gallinarum	Enteritidis	2076_2077	3204651	3205517	-1	YP_002245085	NA
205	Gallinarum	Enteritidis	2115_3347	3256339	3257337	1	YP_002245131	ygjR
206	Gallinarum	Enteritidis	2115_2116	3258831	3260075	1	YP_002245133	ygjU
207	Gallinarum	Enteritidis	2127_3611	3270338	3271701	-1	YP_002245147	tdcG
208	Gallinarum	Enteritidis	3367_3923	3421248	3424360	1	YP_002245290	acrF
209	Gallinarum	Enteritidis	2341_2342	3447295	3449027	1	YP_002245959	yijP
210	Gallinarum	Enteritidis	3389_3390	3508517	3509475	-1	YP_002245905	NA

ID	Query	Subject	Anchor	Query_start	Query_end	Query_strand	Subject_protein	Subject_name
211	Gallinarum	Enteritidis	2374_3391	3518828	3519517	-1	YP_002245895	yiiM
212	Gallinarum	Enteritidis	2376_2377	3525844	3526691	-1	YP_002245891	rhaR
213	Gallinarum	Enteritidis	2380_3633	3531790	3532937	1	YP_002245886	NA
214	Gallinarum	Choleraesuis	2384_3395	3536574	3537594	1	YP_218915	NA
215	Gallinarum	Enteritidis	2393_2394	3550980	3551882	-1	YP_002245865	yihV
216	Gallinarum	Enteritidis	2394_3955	3552960	3553838	1	YP_002245863	yihT
217	Gallinarum	Enteritidis	3965_4148	3608774	3609737	-1	YP_002245986	NA
218	Gallinarum	Choleraesuis	2287_2288	3695203	3695427	-1	YP_218815	rffH
219	Gallinarum	Enteritidis	2415_2416	3735029	3736534	-1	YP_002245756	rbsA
220	Gallinarum	Enteritidis	2419_2420	3742336	3743328	-1	YP_002245751	asnA
221	Gallinarum	Enteritidis	2435_3409	3762472	3763844	1	YP_002245732	NA
222	Gallinarum	Enteritidis	3414_3949	3795047	3797781	-1	YP_002245702	torS
223	Gallinarum	Enteritidis	2478_3426	3852062	3852956	-1	YP_002245648	yicL
224	Gallinarum	Enteritidis	2480_3788	3870266	3871647	1	YP_002245637	yicJ
225	Gallinarum	Enteritidis	2509_2510	3906119	3906927	1	YP_002245603	rfaZ
226	Gallinarum	Enteritidis	2530_3435	3929969	3931625	-1	YP_002245580	lldP
227	Gallinarum	Enteritidis	3439_4143	3954913	3956408	-1	YP_002245562	lyxK
228	Gallinarum	Enteritidis	2541_3441	3964807	3966818	-1	YP_002245552	malS
229	Gallinarum	Enteritidis	2545_2546	3972987	3973982	-1	YP_002245546	yiaH
230	Gallinarum	Enteritidis	2547_2548	3979459	3979944	1	YP_002245539	NA
231	Gallinarum	Enteritidis	3939_3940	3991032	3993559	1	YP_002245526	lpfC
232	Gallinarum	Enteritidis	2558_3445	3997411	3998410	-1	YP_002245522	NA
233	Gallinarum	Enteritidis	2560_2561	4005327	4006299	1	YP_002245516	dppD
234	Gallinarum	Enteritidis	3452_3453	4013424	4014176	1	YP_002245509	yhjQ
235	Gallinarum	Enteritidis	2567_2568	4031108	4033168	1	YP_002245499	yhjG
236	Gallinarum	Enteritidis	2570_3456	4037512	4038110	1	YP_002245495	yhjB
237	Gallinarum	Enteritidis	2610_3463	4097204	4098237	-1	YP_002245439	NA
238	Gallinarum	Enteritidis	2615_2616	4109578	4111712	1	YP_002245428	glgB
239	Gallinarum	Enteritidis	2671_2672	4207733	4209539	1	YP_002245350	kefB
240	Gallinarum	Enteritidis	2715_2716	4239945	4241068	1	YP_002245298	smf

ID	Query	Subject	Anchor	Query_start	Query_end	Query_strand	Subject_protein	Subject_name
241	Gallinarum	Enteritidis	2772_2773	4337509	4339103	1	YP_002246068	yjcC
242	Gallinarum	Choleraesuis	2804_3634	4387045	4389474	1	YP_219170	dmsA
243	Gallinarum	Enteritidis	2817_2818	4406455	4407692	-1	YP_002246128	yjeH
244	Gallinarum	Enteritidis	2820_2821	4410597	4411454	-1	YP_002246132	yjeJ
245	Gallinarum	Enteritidis	2837_2838	4431585	4433131	1	YP_002246153	yjeF
246	Gallinarum	Enteritidis	3493_3495	4450534	4450931	1	YP_002246170	yjfL
247	Gallinarum	Enteritidis	2851_3496	4452830	4454432	1	YP_002246173	aidB
248	Gallinarum	Enteritidis	2882_3499	4489189	4490352	-1	YP_002246211	NA
249	Gallinarum	Enteritidis	2890_3504	4501693	4502433	1	YP_002246225	NA
250	Gallinarum	Enteritidis	2896_2897	4515503	4515949	-1	YP_002246236	NA
251	Gallinarum	Enteritidis	2903_2904	4522317	4523537	-1	YP_002246245	NA
252	Gallinarum	Enteritidis	2951_2952	4631279	4632600	1	YP_002246347	NA
253	Gallinarum	Enteritidis	2963_3521	4649003	4650050	-1	YP_002246363	sthE
254	Gallinarum	Enteritidis	3521_3818	4650666	4653204	-1	YP_002246365	sthB
255	Gallinarum	Choleraesuis	3522_4151	4653250	4653933	-1	YP_219426	sthA
256	Gallinarum	Enteritidis	2972_73	17759	19855	1	YP_002242184	NA
257	Gallinarum	Enteritidis	3872_3873	1833750	1834514	1	YP_002243790	ydiQ
258	Gallinarum	Enteritidis	1385_1386	2136046	2138328	1	YP_002244152	sopA
259	Gallinarum	Enteritidis	2403_2404	3575719	3576215	-1	YP_002245846	yihI
260	Gallinarum	Enteritidis	370_371	545824	546770	-1	YP_002242620	aes
261	Gallinarum	Enteritidis	946_947	1600546	1603568	-1	YP_002243562	srfB
262	Typhi	Typhimurium	198_199	228147	230338	1	NP_459196	fhuA
263	Typhi	Typhimurium	211_212	244959	246261	1	NP_459217	NA
264	Typhi	Typhimurium	3028_3667	396876	399850	1	NP_459353	res
265	Typhi	Typhimurium	297_298	436395	437567	-1	NP_459389	araJ
266	Typhi	Typhimurium	363_364	531966	532481	-1	NP_459476	priC
267	Typhi	Typhimurium	373_374	546660	548311	1	NP_459489	ushA
268	Typhi	Choleraesuis	3053_3672	569227	569708	1	YP_215543	allA
269	Typhi	Choleraesuis	3056_3057	575648	577102	1	YP_215548	allP
270	Typhi	Typhimurium	3067_393	596706	597239	1	NP_459539	fimI

ID	Query	Subject	Anchor	Query_start	Query_end	Query_strand	Subject_protein	Subject_name
271	Typhi	Typhimurium	414_415	633715	634851	1	NP_459581	fepE
272	Typhi	Typhimurium	451_452	678420	679207	1	NP_459623	ybeM
273	Typhi	Typhimurium	3089_563	837955	838660	1	NP_459785	ybhL
274	Typhi	Typhimurium	575_576	855280	856201	1	NP_459803	ybiN
275	Typhi	Typhimurium	3097_657	964573	965531	1	NP_459947	NA
276	Typhi	Typhimurium	668_669	983712	984944	1	NP_459961	ycaQ
277	Typhi	Typhimurium	3100_3101	1004375	1005590	1	NP_459977	NA
278	Typhi	Typhimurium	3111_703	1081208	1081755	1	NP_460055	NA
279	Typhi	Typhimurium	37_718	1099596	1101061	1	NP_460075	hpaE
280	Typhi	Typhimurium	755_756	1155708	1156280	-1	NP_460129	NA
281	Typhi	Typhimurium	794_795	1197551	1199726	-1	NP_460174	fhuE
282	Typhi	Typhimurium	838_839	1254184	1254642	-1	NP_460714	ychJ
283	Typhi	Typhimurium	847_848	1267159	1268066	1	NP_460703	oppC
284	Typhi	Typhimurium	916_917	1360852	1362226	1	NP_460614	dbpA
285	Typhi	Typhimurium	945_946	1419165	1419292	1	NP_460555	ydcX
286	Typhi	Typhimurium	962_963	1481216	1483018	1	NP_460498	NA
287	Typhi	Typhimurium	969_970	1490098	1490174	-1	NP_460485	yneH
288	Typhi	Typhimurium	3176_988	1512377	1512686	1	NP_460460	ynfD
289	Typhi	Typhimurium	1079_3188	1653541	1655314	-1	NP_460351	ttrS
290	Typhi	Typhimurium	1080_3713	1660631	1661849	1	NP_460347	orf408
291	Typhi	Typhimurium	1081_3713	1662657	1663345	1	NP_460345	orf32
292	Typhi	Typhimurium	1084_1085	1667459	1668459	-1	NP_460340	ynhG
293	Typhi	Typhimurium	1145_1146	1744888	1745772	1	NP_460255	yeaD
294	Typhi	Typhimurium	1195_1196	1816673	1818385	1	NP_460752	treA
295	Typhi	Typhimurium	3209_3210	1873610	1874331	-1	NP_460811	sopE2
296	Typhi	Typhimurium	1279_1280	1969479	1971350	1	NP_460867	NA
297	Typhi	Choleraesuis	1312_43	2009890	2011094	-1	YP_216948	fliB
298	Typhi	Typhimurium	1339_3224	2035491	2036402	-1	NP_460940	yedI
299	Typhi	Typhimurium	1354_1355	2059701	2060347	-1	NP_460968	cbiM
300	Typhi	Typhimurium	1361_3228	2067551	2068030	-1	NP_460978	cbiC

ID	Query	Subject	Anchor	Query_start	Query_end	Query_strand	Subject_protein	Subject_name
301	Typhi	Typhimurium	3232_3233	2081704	2081978	1	NP_460994	pduN
302	Typhi	Typhimurium	1406_1407	2153034	2154247	-1	NP_461057	wcaD
303	Typhi	Typhimurium	1408_1409	2155927	2156777	-1	NP_461060	wcaA
304	Typhi	Choleraesuis	1411_1412	2160688	2160932	1	YP_217107	NA
305	Typhi	Typhimurium	1426_3242	2199596	2200495	1	NP_461084	yegS
306	Typhi	Typhimurium	1455_1456	2251323	2252841	-1	NP_461134	mglA
307	Typhi	Typhimurium	1462_1463	2261543	2262865	1	NP_461143	NA
308	Typhi	Typhimurium	1591_1592	2447375	2447924	-1	NP_461323	yfcM
309	Typhi	Typhimurium	1625_1626	2505709	2507437	1	NP_461367	ptsI
310	Typhi	Typhimurium	1649_1650	2535426	2535715	-1	NP_461400	eutM
311	Typhi	Enteritidis	1673_3288	2568948	2570135	-1	YP_002244556	NA
312	Typhi	Typhimurium	1687_3290	2612859	2615051	-1	NP_461452	sinH
313	Typhi	Typhimurium	1698_3291	2627135	2629539	-1	NP_461465	NA
314	Typhi	Typhimurium	1712_1713	2653445	2654430	-1	NP_461486	NA
315	Typhi	Typhimurium	1788_1789	2784276	2785289	-1	NP_461709	nixA
316	Typhi	Typhimurium	1798_1799	2798046	2798495	-1	NP_461721	ygaU
317	Typhi	Typhimurium	3309_3310	2802627	2803961	-1	NP_461729	NA
318	Typhi	Typhimurium	1807_1808	2808847	2810048	1	NP_461735	proV
319	Typhi	Choleraesuis	1916_3752	2948970	2949557	1	YP_217876	stfA
320	Typhi	Typhimurium	3335_3336	3041128	3041988	1	NP_461941	yohM
321	Typhi	Typhimurium	2037_3338	3129168	3131292	-1	NP_462030	speC
322	Typhi	Typhimurium	2060_2061	3170215	3171858	-1	NP_462067	NA
323	Typhi	Typhimurium	2070_2071	3181088	3181915	1	NP_462080	dkgA
324	Typhi	Typhimurium	2079_2080	3200413	3200993	1	NP_462094	mdaB
325	Typhi	Typhimurium	2112_2113	3243758	3245775	1	NP_462133	fadH
326	Typhi	Typhimurium	2128_2129	3267539	3268869	-1	NP_462157	tdcC
327	Typhi	Typhimurium	2198_2199	3360298	3360977	-1	NP_462247	nanE
328	Typhi	Typhimurium	2350_3378	3636740	3638529	1	NP_462979	NA
329	Typhi	Typhimurium	2374_3778	3679220	3680202	1	NP_462935	NA
330	Typhi	Typhimurium	2379_3394	3689433	3690259	1	NP_462926	rhaD

ID	Query	Subject	Anchor	Query_start	Query_end	Query_strand	Subject_protein	Subject_name
331	Typhi	Typhimurium	3401_3402	3723230	3724471	1	NP_462892	NA
332	Typhi	Typhimurium	2411_2412	3750269	3751696	1	NP_462786	yieO
333	Typhi	Typhimurium	2461_2462	3841645	3841992	1	NP_462706	yidH
334	Typhi	Typhimurium	2466_2467	3849242	3850075	-1	NP_462698	NA
335	Typhi	Typhimurium	4073_4074	3868106	3868966	1	NP_462679	gatY
336	Typhi	Typhimurium	3420_3421	3873448	3876247	1	NP_462672	NA
337	Typhi	Typhimurium	2479_3428	3887569	3888047	1	NP_462661	cigR
338	Typhi	Typhimurium	3429_3430	3891261	3894130	-1	NP_462656	misL
339	Typhi	Typhimurium	2488_2489	3915457	3917141	1	NP_462639	ligB
340	Typhi	Typhimurium	2525_2526	3955101	3956411	-1	NP_462598	NA
341	Typhi	Typhimurium	2545_2546	4004508	4005502	-1	NP_462558	yiaH
342	Typhi	Typhimurium	3445_3446	4025298	4026622	1	NP_462533	NA
343	Typhi	Typhimurium	2584_3459	4093033	4094155	1	NP_462486	yhhJ
344	Typhi	Typhimurium	2598_3800	4110398	4111495	1	NP_462468	livJ
345	Typhi	Typhimurium	2664_2665	4211189	4213276	-1	NP_462370	yhfK
346	Typhi	Typhimurium	2668_2669	4215901	4216888	-1	NP_462365	yheT
347	Typhi	Enteritidis	2804_2805	4399438	4400064	1	YP_002246110	dmsB2
348	Typhi	Enteritidis	4085_4086	4703505	4704159	1	YP_002246273	NA
349	Typhi	Typhimurium	3516_3813	4738848	4740219	1	NP_463378	NA
350	Typhi	Typhimurium	2959_2960	4793252	4793587	-1	NP_463440	yjjX
351	Typhi	Typhimurium	2963_3521	4799424	4800508	-1	NP_463447	sthE
352	Typhi	Typhimurium	76_77	25807	28428	1	NP_459028	bcfC
353	Typhi	Typhimurium	314_315	461599	462049	1	NP_459410	nrdR
354	Typhi	Typhimurium	1235_1236	1860238	1861610	1	NP_460799	NA
355	Typhi	Typhimurium	1746_3301	2699857	2700754	-1	NP_461579	yfiE
356	Typhi	Typhimurium	2126_3350	3260857	3262187	-1	NP_462153	yhaO
357	Typhi	Typhimurium	3418_3785	3869643	3870001	-1	NP_462676	NA
358	Typhi	Typhimurium	3428_3429	3889904	3890691	1	NP_462658	marT
359	Typhi	Typhimurium	3521_3818	4801124	4803660	-1	NP_463449	sthB
360	Typhi	Typhimurium	496_497	739365	741433	-1	NP_459686	speF

ID	Query	Subject	Anchor	Query_start	Query_end	Query_strand	Subject_protein	Subject_name
361	Typhi	Typhimurium	3237_3238	2140648	2141926	-1	NP_461046	wcaK
362	Typhi	Typhimurium	2562_2563	4045980	4049523	1	NP_462517	yhjL
363	Typhi	Typhimurium	1385_1386	2098342	2100684	1	NP_461011	sopA
364	Typhi	Enteritidis	478_479	713980	714047	-1	YP_002242788	NA
365	Typhi	Enteritidis	1686_4049	2592456	2597379	-1	YP_002244574	shdA
366	Typhimurium	Enteritidis	239_240	281688	282393	1	YP_002242410	NA
367	Typhimurium	Choleraesuis	261_3015	359074	360206	1	YP_215302	ykfJ
368	Typhimurium	Typhi	549_550	854969	856653	1	NP_455332	hutU
369	Typhimurium	Typhi	732_733	1207597	1211558	-1	NP_455618	putA
370	Typhimurium	Typhi	982_983	1584546	1585925	1	NP_455973	NA
371	Typhimurium	Enteritidis	3139_3698	1759222	1759587	1	YP_002243470	NA
372	Typhimurium	Choleraesuis	1347_1348	2096074	2096245	-1	YP_217009	NA
373	Typhimurium	Typhi	1930_1931	3126871	3128188	1	NP_457370	fucP
374	Typhimurium	Choleraesuis	2165_2166	3471407	3472371	-1	YP_218227	rhbE
375	Typhimurium	Enteritidis	3931_4141	3693941	3695274	1	YP_002245420	NA
376	Typhimurium	Choleraesuis	3950_3951	4031780	4032396	-1	YP_218733	dgoA
377	Typhimurium	Choleraesuis	2287_2288	4132779	4133003	1	YP_218815	rffH
378	Typhimurium	Enteritidis	2895_3810	4696802	4698219	-1	YP_002246233	treB

ID	Subject_product	Gene_length	Percent_alignable
1	fimbrial usher	2622	100%
2	putative sodium galactoside symporter	1374	100%
3	lipoprotein involved with copper homeostasis and adhesion	702	99%
4	putative fimbrial chaparone	759	100%
5	putative cation efflux system protein	3168	100%
6	putative cation transport ATPase	2289	100%
7	DNA-binding transcriptional repressor AcrR	654	100%
8	putative periplasmic protein	828	100%
9	glyoxylate carboligase	1782	100%
10	hypothetical protein	312	100%
11	putative dehydrogenase	1239	100%
12	ribonuclease I	807	100%
13	palmitoyl transferase	573	100%
14	putative molecular chaperone	1473	99%
15	citrate utilization protein b	1140	100%
16	transcriptional regulator	984	100%
17	putative SAM-dependent methyltransferase	927	100%
18	nitroreductase A	723	100%
19	cell division protein MukB	4467	100%
20	ABC transporter ATPase component	1908	100%
21	putative sodium/glucose cotransporter	1497	100%
22	putative sialic acid transporter	1281	100%
23	putative virulence protein	924	100%
24	O-antigen polymerase	1224	0%
25	putative oxidoreductase	1041	100%
26	putative voltage-gated ClC-type chloride channel ClcB	1254	100%
27	putative transcriptional regulator	873	100%
28	formate dehydorgenase-N alpha subunit	3048	100%
29	nitrate reductase 2 alpha subunit	3741	100%
30	putative DNA-binding transcriptional regulator	666	100%

ID	Subject_product	Gene_length	Percent_alignable
31	putative nucleoside-diphosphate-sugar pyrophosphorylase	981	100%
32	putative carboxylesterase	1509	100%
33	putative thiol peroxidase	558	100%
34	invasin-like protein	1983	98%
35	putative 2'-hydroxyisoflavone reductase	930	100%
36	hydrogenase 1 large subunit	1794	100%
37	copper homeostasis protein CutC	747	100%
38	hypothetical protein	912	100%
39	putative lipoprotein	531	100%
40	galactose/methyl galaxtoside transporter ATP-binding protein	1521	100%
41	putative inner membrane protein	648	100%
42	putative carboxysome structural protein	495	100%
43	ethanolamine ammonia-lyase small subunit	897	100%
44	nitrate/nitrite sensor protein NarQ	1701	100%
45	host colonisation factor (ShdA)	6066	100%
46	chaperone protein HscA	1851	100%
47	hypothetical protein	1221	100%
48	putative D-glucarate dehydratase	1341	100%
49	putative hydrolase	954	100%
50	putative mannitol dehydrogenase	1473	100%
51	putative glutathione S-transferase	987	100%
52	dihydropteroate synthase	849	100%
53	putative signal transduction protein	2100	100%
54	30S ribosomal protein S13	357	100%
55	putative dehydratase	1716	100%
56	high-affinity branched-chain amino acid transporter	1110	100%
57	L-ribulose-5-phosphate 4-epimerase	696	100%
58	putative cytoplasmic protein	1956	100%
59	putative permease	1311	100%
60	putative selenocysteine synthase	1110	95%

ID	Subject_product	Gene_length	Percent_alignable
61	ribonucleoside transporter	1194	100%
62	regulatory protein UhpC	1329	100%
63	TMAO reductase system periplasmic protein TorT	1041	100%
64	hypothetical protein	258	100%
65	DNA-binding transcriptional regulator YidZ	960	97%
66	acetolactate synthase 2 catalytic subunit	1647	100%
67	putative arylsulfatase regulator	1344	100%
68	potassium transporter	1452	100%
69	putative acyltransferase	909	100%
70	putative isomerase	1242	86%
71	formate dehydrogenase alpha subunit	3051	100%
72	putative ABC-type aldose transport system ATPase component	1536	100%
73	putative 5'-nucleotidase/2',3'-cyclic phosphodiesterase	1557	100%
74	putative formate acetyltransferase 2	2298	100%
75	putative cytoplasmic protein	345	100%
76	putative cytoplasmic protein	189	0%
77	formate dehydrogenase	2148	100%
78	putative cytoplasmic protein	267	100%
79	putative phage shock protein A	699	100%
80	putative reductase	849	100%
81	putative metallo-dependent hydrolase	1164	100%
82	putative exochitinase	2100	100%
83	putative hydrolas	1173	99%
84	putative inner membrane protein	387	100%
85	putative inner membrane protein	561	100%
86	suppression of copper sensitivity protein	1887	100%
87	hypothetical protein	540	94%
88	nickel/cobalt efflux protein RcnA	867	99%
89	putative phosphotransferase system fructose-specific component IIB	1374	98%
90	putative permease	699	100%

ID	Subject_product	Gene_length	Percent_alignable
91	multidrug efflux system protein MdtE	1413	100%
92	hypothetical protein	447	6%
93	hypothetical protein	576	11%
94	hypothetical protein	1203	100%
95	putative aldo/keto reductase	990	100%
96	hypothetical protein	1494	100%
97	hypothetical protein	1239	100%
98	nitroreductase A	723	100%
99	glucose-1-phosphatase/inositol phosphatase	1242	100%
100	hypothetical protein	1551	100%
101	lipid kinase	900	100%
102	putative transport protein	1290	100%
103	putative outer membrane protein	7308	100%
104	intimin-like protein	2193	93%
105	hypothetical protein	810	100%
106	putative hydrolase	954	100%
107	major facilitator superfamily nucleoside transporter	1257	100%
108	putative transmembrane efflux protein	1428	100%
109	glucose-1-phosphate thymidylyltransferase	882	26%
110	putative membrane transport protein	1422	100%
111	putative inner membrane lipoprotein	1032	99%
112	peptidase E	705	100%
113	putative anaerobic dimethyl sulfoxide reductase, subunit A	2430	100%
114	exported protein	489	100%
115	hypothetical protein	714	100%
116	putative hydroxymethyltransferase	2997	100%
117	putative transport protein	1374	100%
118	putative crotonobetaine/carnitine-CoA ligase	1554	100%
119	glucose dehydrogenase	2391	100%
120	putativie fimbrial usher. putativie fimbrial usher	2547	100%

ID	Subject_product	Gene_length	Percent_alignable
121	putative PTS system IIA component	441	98%
122	minor fimbrial subunit stff	477	100%
123	putative outer membrane protein	840	100%
124	salmonella atypical fimbria outer membrane usher	2511	99%
125	outer membrane phosphoporin protein E	1053	100%
126	outer membrane fimbrial usher protein	2562	100%
127	type III restriction-modification system enzyme (StyLTI) modification methylase	1959	100%
128	hypothetical protein	456	43%
129	hypothetical protein	399	100%
130	putative multidrug transporter membrane\ATP-binding components	1773	100%
131	thioredoxin-like protein	855	100%
132	DNA-binding transcriptional activator AllS	927	100%
133	membrane protein FdrA	1665	100%
134	threonine-phosphate decarboxylase	1095	100%
135	putative sigma-54 dependent transcriptional regulator	1929	100%
136	ATP-dependent DNA helicase DinG	2145	100%
137	glutamine ABC transporter periplasmic protein	747	100%
138	23S rRNA methyluridine methyltransferase	1128	100%
139	macrolide transporter subunit MacA	1119	100%
140	putative transcriptional regulator	639	62%
141	exported protein	1155	100%
142	hypothetical protein	921	100%
143	hypothetical protein	327	100%
144	methyl-accepting chemotaxis protein II	1662	100%
145	putative membrane transport protein	1374	91%
146	hypothetical protein	459	100%
147	hydrogenase-1 operon protein HyaF	849	100%
148	hypothetical protein	1134	100%
149	hypothetical protein	1374	100%
150	probable pyruvate-flavodoxin oxidoreductase	3525	100%

ID	Subject_product	Gene_length	Percent_alignable
151	secreted effector protein	951	100%
152	putative hydrolase	2529	100%
153	hypothetical protein	948	100%
154	putative membrane transport protein	1380	100%
155	putative dimethyl sulphoxide reductase subunit A	2442	90%
156	putative membrane transport protein	1254	100%
157	putative oxidoreductase	897	100%
158	Orf 245 protein	738	100%
159	hypothetical protein	1002	100%
160	hypothetical protein	1443	99%
161	arginine succinyltransferase	1035	45%
162	glutamate dehydrogenase	1344	100%
163	Putative metabolite transport protein	1359	100%
164	putative oxidoreductase	1044	100%
165	hypothetical protein	1134	100%
166	cold shock protein (CspH)	213	95%
167	hypothetical protein	540	96%
168	flagellar hook-associated protein FlgK	1662	100%
169	flagellar basal body P-ring protein	1098	100%
170	hypothetical protein	648	100%
171	cobalt transporter ATP-binding subunit	816	100%
172	pdu/cob regulatory protein PocR	912	98%
173	propanediol utilization protein	1833	100%
174	propanediol utilization protein	1011	96%
175	multidrug efflux system protein MdtE	1413	100%
176	putative outer membrane usher protein	2394	100%
177	putative lipoprotein	531	100%
178	acetoin dehydrogenase	762	100%
179	putative transcriptional regulator	909	86%
180	galactose/methyl galaxtoside transporter ATP-binding protein	1521	100%

ID	Subject_product	Gene_length	Percent_alignable
181	hypothetical protein	1557	100%
182	hypothetical protein	648	100%
183	putative amino acid transporter	1422	100%
184	putative transcriptional regulator	1428	100%
185	hypothetical protein	2190	100%
186	hypothetical protein	576	100%
187	host colonisation factor (ShdA)	6066	100%
188	putative outer membrane protein	7308	100%
189	intimin-like protein	2193	100%
190	anaerobic sulfite reductase subunit A	1044	100%
191	putative inner membrane protein	987	100%
192	putative type I secretion protein, ATP-binding protein	2181	100%
193	ferric enterochelin esterase	1245	100%
194	exported protein	1053	100%
195	putative cation transporter	1014	100%
196	hydroxyglutarate oxidase	1269	99%
197	DNA binding protein, nucleoid-associated	402	100%
198	multidrug resistance protein B	1539	100%
199	hypothetical protein	2664	100%
200	2-deoxy-D-gluconate 3-dehydrogenase	762	100%
201	hypothetical protein	1008	100%
202	ornithine decarboxylase	2136	100%
203	exported protein	516	100%
204	possible AraC-family transcriptional regulator	867	100%
205	possible oxidoreductase	999	100%
206	serine/threonine transporter SstT	1245	100%
207	L-serine dehydratase	1365	100%
208	acriflavin resistance protein F	3114	100%
209	hypothetical protein	1734	100%
210	aminoimidazole riboside kinase	960	100%

ID	Subject_product	Gene_length	Percent_alignable
211	hypothetical protein	690	100%
212	transcriptional activator RhaR	849	100%
213	alcohol dehydrogenase	1149	100%
214	putative inner membrane lipoprotein	1032	99%
215	putative sugar kinase	903	100%
216	putative aldolase	879	100%
217	hypothetical protein	1011	95%
218	glucose-1-phosphate thymidylyltransferase	882	26%
219	D-ribose transporter ATP binding protein	1506	100%
220	asparagine synthetase AsnA	993	100%
221	probable PTS system permease	1374	100%
222	hybrid sensory histidine kinase TorS	2736	100%
223	hypothetical protein	903	99%
224	putative transporter	1383	100%
225	lipopolysaccharide core biosynthesis protein	810	100%
226	L-lactate permease	1656	100%
227	putative L-xylulose kinase	1497	100%
228	periplasmic alpha-amylase precursor	2028	99%
229	hypothetical protein	996	100%
230	putative acetyltransferase	486	100%
231	outer membrane usher protein (LpfC)	2529	100%
232	putative lacI-family transcriptional regulator	1008	99%
233	dipeptide transporter ATP-binding subunit	984	99%
234	cell division protein	753	100%
235	hypothetical protein	2061	100%
236	hypothetical luxR-family transcriptional regulator	603	99%
237	puative phophotriesterase	1035	100%
238	glycogen branching enzyme	2187	98%
239	glutathione-regulated potassium-efflux system protein KefB	1806	100%
240	DNA protecting protein DprA	1125	100%

ID	Subject_product	Gene_length	Percent_alignable
241	hypothetical protein	1602	100%
242	putative anaerobic dimethyl sulfoxide reductase, subunit A	2430	100%
243	inner membrane protein YjeH	1242	100%
244	hypothetical protein	858	100%
245	hypothetical protein	1548	100%
246	hypothetical protein	399	100%
247	isovaleryl CoA dehydrogenase	1623	99%
248	putative metallo-dependent hydrolase	1164	100%
249	hypothetical protein	741	100%
250	hypothetical protein	447	100%
251	arginine deiminase	1221	100%
252	hypothetical protein	1329	100%
253	putative fimbrial subunit	1086	97%
254	Outer membrane fimbrial usher protein	2538	100%
255	putative fimbrial chaparone protein	684	100%
256	putative chitinase	2100	100%
257	putative electron transfer flavoprotein YdiQ	765	100%
258	secreted protein SopA	2349	97%
259	hypothetical protein	516	96%
260	acetyl esterase	972	97%
261	putative virulence effector protein	2982	100%
262	ferrichrome outer membrane transporter	2190	100%
263	putative inner membrane protein	1302	100%
264	DNA restriction enzyme	2976	100%
265	MFS transport protein AraJ	1173	100%
266	primosomal replication protein N"	516	100%
267	bifunctional UDP-sugar hydrolase/5'-nucleotidase periplasmic precursor	1653	100%
268	ureidoglycolate hydrolase	483	100%
269	allantoin permease	1455	100%
270	fimbrial protein	534	100%

ID	Subject_product	Gene_length	Percent_alignable
271	ferric enterobactin transport protein FepE	1137	100%
272	putative hydrolase	789	100%
273	putative permease	705	100%
274	putative SAM-dependent methyltransferase	927	99%
275	SopD-like protein	960	100%
276	putative cytoplasmic protein	1233	100%
277	diaminopropionate ammonia-lyase	1215	100%
278	putative outer membrane protein	549	100%
279	4-hydroxyphenylacetate catabolism	1467	100%
280	putative inner membrane protein	573	100%
281	ferric-rhodotorulic acid outer membrane transporter	2175	100%
282	hypothetical protein	459	100%
283	oligopeptide transport protein	909	100%
284	ATP-dependent RNA helicase DbpA	1374	100%
285	putative inner membrane protein	264	49%
286	putative hydrogenase-1 large subunit	1803	100%
287	glutaminase	927	8%
288	putative outer membrane protein	309	100%
289	sensory histidine kinase	1779	100%
290	putative regulatory protein	1227	99%
291	putative proline iminopeptidase	894	77%
292	hypothetical protein	1002	100%
293	aldose 1-epimerase	885	100%
294	trehalase	1713	100%
295	type III-secreted effector protein	723	100%
296	putative penicillin-binding protein	1872	100%
297	N-methylation of lysine residues in flagellin	1206	100%
298	hypothetical protein	912	100%
299	cobalt transport protein CbiM	648	100%
300	cobalt-precorrin-8X methylmutase	633	76%

ID	Subject_product	Gene_length	Percent_alignable
301	polyhedral body protein	276	100%
302	putative colanic acid biosynthesis protein	1215	100%
303	putative glycosyl transferase	843	100%
304	hypothetical protein	246	100%
305	lipid kinase	900	100%
306	galactose/methyl galaxtoside transporter ATP-binding protein	1521	100%
307	putative regulatory protein	1323	100%
308	putative cytoplasmic protein	549	100%
309	phosphoenolpyruvate-protein phosphotransferase	1728	100%
310	putative detox protein	291	100%
311	conserved regulatory protein	1188	100%
312	intimin-like protein	2193	100%
313	putative anaerobic dimethylsulfoxide reductase	2406	100%
314	putative inner membrane protein	987	100%
315	putative nickel transporter	1014	100%
316	LysM domain/BON superfamily protein	450	100%
317	putative regulatory protein	1335	100%
318	glycine betaine transporter ATP-binding subunit	1203	100%
319	putative fimbrial subunit	588	100%
320	nickel/cobalt efflux protein RcnA	867	99%
321	ornithine decarboxylase	2136	100%
322	putative methyl-accepting chemotaxis protein	1644	100%
323	2,5-diketo-D-gluconate reductase A	828	100%
324	NADPH-specific quinone oxidoreductase	582	100%
325	2,4-dieonyl-CoA reductase	2019	100%
326	threonine/serine transporter TdcC	1332	100%
327	N-acetylmannosamine-6-phosphate 2-epimerase	690	99%
328	putative arylsulfate sulfotransferase	1791	100%
329	putative periplasmic dicarboxylate-binding protein	984	100%
330	rhamnulose-1-phosphate aldolase	828	100%

ID	Subject_product	Gene_length	Percent_alignable
331	coproporphyrinogen III oxidase	1242	100%
332	putative tranport protein	1428	100%
333	putative inner membrane protein	348	100%
334	integral membrane protein	834	100%
335	putative fructose-1,6-bisphosphate aldolase	861	100%
336	putative transcriptional regulator	2799	100%
337	putative inner membrane protein	480	100%
338	putative autotransporter	2868	100%
339	NAD-dependent DNA ligase LigB	1686	100%
340	putative permease	1311	100%
341	putative inner membrane protein	996	100%
342	putative xanthine permease	1326	100%
343	putative ABC transport protein	1125	100%
344	high-affinity branched-chain amino acid transporter	1098	100%
345	putative inner membrane protein	2088	100%
346	putative hydrolase	1068	93%
347	anaerobic dimethyl sulfoxide reductase chain B	627	100%
348	Thiol:disulfide interchange protein	654	100%
349	putative NAD-dependent aldehyde dehydrogenase	1371	100%
350	NTPase	516	65%
351	putative major fimbrial subunit	1086	100%
352	fimbrial usher	2622	100%
353	transcriptional regulator NrdR	450	100%
354	putative transport protein	1374	100%
355	putative transcriptional regulator	900	100%
356	putative transport protein	1332	100%
357	putative cytoplasmic protein	360	100%
358	putative transcriptional regulator	789	100%
359	putative fimbrial usher protein	2538	100%
360	ornithine decarboxylase	2199	93%

ID	Subject_product	Gene_length	Percent_alignable
361	putative pyruvyl transferase	1281	100%
362	cellulose synthase subunit BcsC	3543	100%
363	secreted effector protein	2349	100%
364	hypothetical protein	576	11%
365	host colonisation factor (ShdA)	6066	81%
366	lipoprotein involved with copper homeostasis and adhesion	702	100%
367	hypothetical protein	1140	99%
368	urocanate hydratase	1686	100%
369	trifunctional transcriptional regulator/proline dehydrogenase/pyrroline-5-carboxylate dehydrogenase	3963	100%
370	putative membrane transport protein	1380	100%
371	hypothetical protein	366	100%
372	hypothetical protein	171	100%
373	L-fucose transporter	1317	100%
374	hypothetical protein	966	100%
375	putative MFS-family membrane transport protein	1335	100%
376	2-dehydro-3-deoxy-6-phosphogalactonate aldolase	618	100%
377	glucose-1-phosphate thymidylyltransferase	882	26%
378	trehalose(maltose)-specific PTS system components IIBC	1419	100%

ID	Percent_identity	Num_deleletion	Num_insertion	Num_pre_stop
1	99%	0	0	1
2	100%	1	0	0
3	100%	1	0	0
4	98%	0	1	0
5	99%	0	0	1
6	99%	0	1	0
7	100%	0	0	1
8	98%	1	0	0
9	99%	0	1	0
10	99%	0	1	0
11	99%	0	1	0
12	98%	0	1	0
13	98%	0	0	1
14	98%	0	0	1
15	100%	0	0	1
16	99%	0	1	0
17	99%	0	1	0
18	99%	0	0	1
19	99%	0	1	0
20	100%	0	0	1
21	99%	1	0	0
22	100%	0	1	0
23	99%	0	1	0
24	0%	1	0	0
25	99%	0	0	1
26	99%	0	0	1
27	99%	0	0	1
28	99%	0	0	1
29	99%	0	0	1
30	99%	0	0	1

ID	Percent_identity	Num_deleletion	Num_insertion	Num_pre_stop
31	98%	0	1	0
32	99%	0	0	1
33	99%	0	0	1
34	98%	1	0	0
35	99%	0	1	0
36	99%	0	1	0
37	99%	1	0	0
38	99%	0	0	1
39	100%	1	0	0
40	99%	1	0	0
41	100%	1	0	0
42	99%	0	0	1
43	98%	1	0	0
44	99%	0	0	1
45	98%	0	1	0
46	99%	1	0	0
47	100%	0	0	1
48	99%	0	0	1
49	99%	0	1	0
50	99%	0	0	1
51	99%	0	0	1
52	99%	0	1	0
53	99%	0	0	1
54	100%	0	1	0
55	99%	0	0	1
56	99%	0	0	1
57	98%	0	0	1
58	98%	0	0	1
59	99%	0	0	1
60	99%	1	0	0

ID	Percent_identity	Num_deleletion	Num_insertion	Num_pre_stop
61	99%	1	0	0
62	98%	0	0	1
63	99%	0	0	1
64	100%	1	0	0
65	99%	1	0	0
66	99%	1	0	0
67	99%	1	0	0
68	99%	0	0	1
69	99%	1	0	0
70	99%	1	0	0
71	99%	0	0	1
72	100%	1	0	0
73	99%	1	0	0
74	100%	1	0	0
75	97%	0	0	1
76	0%	1	0	0
77	99%	0	0	1
78	98%	0	0	1
79	100%	0	1	0
80	99%	1	0	0
81	99%	1	0	0
82	100%	1	0	1
83	99%	1	1	0
84	100%	1	1	0
85	100%	1	1	0
86	99%	0	0	2
87	100%	1	0	1
88	99%	1	1	0
89	98%	2	0	0
90	100%	2	0	0

ID	Percent_identity	Num_deleletion	Num_insertion	Num_pre_stop
91	99%	3	0	0
92	81%	4	0	0
93	77%	7	0	1
94	100%	0	1	0
95	100%	1	0	0
96	100%	0	0	1
97	100%	0	0	1
98	100%	0	0	1
99	100%	0	0	1
100	100%	1	0	0
101	100%	0	1	0
102	100%	0	0	1
103	99%	0	0	1
104	99%	1	0	0
105	100%	0	1	0
106	100%	0	0	1
107	99%	1	0	0
108	100%	1	0	0
109	100%	1	0	0
110	100%	1	0	0
111	96%	1	0	0
112	100%	0	0	1
113	99%	0	0	1
114	100%	0	1	0
115	100%	0	0	1
116	99%	0	0	1
117	100%	1	0	0
118	100%	0	0	1
119	99%	0	0	1
120	99%	0	0	1

ID	Percent_identity	Num_deleletion	Num_insertion	Num_pre_stop
121	100%	1	0	0
122	95%	0	0	1
123	87%	0	0	1
124	99%	1	0	0
125	100%	0	0	1
126	100%	0	0	0
127	100%	0	1	0
128	100%	1	0	0
129	100%	1	0	0
130	100%	0	0	1
131	100%	0	0	1
132	100%	1	0	0
133	100%	0	0	1
134	100%	0	0	1
135	100%	0	0	1
136	100%	0	0	1
137	100%	1	0	0
138	100%	0	0	1
139	100%	1	0	0
140	100%	1	0	0
141	100%	1	0	0
142	100%	1	0	0
143	100%	1	0	0
144	100%	1	0	0
145	100%	1	0	0
146	100%	0	0	1
147	100%	0	0	1
148	100%	0	1	0
149	100%	1	0	0
150	100%	0	1	0

ID	Percent_identity	Num_deleletion	Num_insertion	Num_pre_stop
151	100%	1	0	0
152	100%	0	1	0
153	100%	0	1	0
154	100%	0	0	1
155	100%	1	0	0
156	100%	0	0	1
157	100%	0	0	1
158	100%	0	0	1
159	100%	0	0	1
160	100%	1	0	0
161	100%	1	0	0
162	100%	0	0	1
163	100%	1	0	0
164	100%	0	1	0
165	100%	1	0	0
166	100%	1	0	0
167	100%	1	0	0
168	100%	0	0	1
169	100%	0	0	1
170	100%	1	0	0
171	100%	1	0	0
172	100%	1	0	0
173	100%	0	1	0
174	100%	1	0	0
175	100%	1	0	0
176	100%	0	1	0
177	100%	1	0	0
178	100%	0	1	0
179	100%	1	0	0
180	100%	1	0	0

ID	Percent_identity	Num_deleletion	Num_insertion	Num_pre_stop
181	100%	0	0	1
182	100%	0	1	0
183	100%	0	0	1
184	100%	1	0	0
185	100%	1	0	0
186	100%	0	0	1
187	100%	1	0	0
188	99%	0	1	0
189	99%	0	1	0
190	100%	0	1	0
191	100%	1	0	0
192	100%	1	0	0
193	100%	0	1	0
194	100%	0	0	1
195	100%	1	0	0
196	100%	1	0	0
197	100%	0	0	1
198	100%	1	0	0
199	100%	0	0	1
200	100%	0	0	1
201	100%	0	0	1
202	99%	1	0	0
203	100%	0	1	0
204	100%	0	0	1
205	100%	0	0	1
206	100%	0	0	1
207	100%	1	0	0
208	100%	1	0	0
209	100%	1	0	0
210	100%	1	0	0

ID	Percent_identity	Num_deleletion	Num_insertion	Num_pre_stop
211	99%	0	0	1
212	100%	1	0	0
213	100%	1	0	0
214	96%	1	0	0
215	100%	0	0	1
216	100%	0	0	1
217	100%	1	0	0
218	100%	1	0	0
219	100%	0	0	1
220	100%	0	0	1
221	100%	1	0	0
222	100%	1	0	0
223	100%	1	0	0
224	100%	1	0	0
225	100%	1	0	0
226	100%	0	1	0
227	100%	1	0	0
228	100%	1	0	0
229	100%	0	0	1
230	100%	0	0	1
231	100%	1	0	0
232	100%	1	0	0
233	100%	1	0	0
234	100%	0	0	1
235	100%	0	0	1
236	100%	1	0	0
237	100%	1	0	0
238	100%	1	0	0
239	100%	0	1	0
240	100%	1	0	0

ID	Percent_identity	Num_deleletion	Num_insertion	Num_pre_stop
241	100%	1	0	0
242	99%	0	0	1
243	100%	1	0	0
244	100%	0	0	1
245	100%	1	0	0
246	100%	1	0	0
247	100%	1	0	0
248	100%	0	0	1
249	100%	0	0	1
250	100%	0	0	1
251	100%	0	0	1
252	100%	1	0	0
253	100%	1	0	0
254	100%	0	1	0
255	99%	0	0	1
256	100%	1	1	0
257	100%	0	0	2
258	100%	1	0	1
259	100%	2	0	0
260	100%	1	0	2
261	100%	0	2	1
262	99%	0	1	0
263	98%	0	1	0
264	99%	1	0	0
265	98%	0	0	1
266	98%	0	0	1
267	99%	1	0	0
268	99%	1	0	0
269	98%	0	0	1
270	98%	0	0	1

ID	Percent_identity	Num_deleletion	Num_insertion	Num_pre_stop
271	98%	0	0	1
272	99%	1	0	0
273	98%	0	1	0
274	99%	0	1	0
275	97%	1	0	0
276	98%	0	0	1
277	98%	0	1	0
278	98%	1	0	0
279	99%	1	0	0
280	98%	0	0	1
281	98%	0	1	0
282	99%	0	0	1
283	99%	1	0	0
284	99%	0	1	0
285	99%	1	0	0
286	99%	0	0	1
287	97%	1	0	0
288	99%	0	1	0
289	99%	1	0	0
290	99%	1	0	0
291	99%	1	0	0
292	99%	1	0	0
293	99%	0	0	1
294	98%	0	0	1
295	98%	1	0	0
296	99%	0	0	1
297	97%	1	0	0
298	99%	0	0	1
299	99%	1	0	0
300	99%	1	0	0

ID	Percent_identity	Num_deleletion	Num_insertion	Num_pre_stop
301	99%	1	0	0
302	99%	1	0	0
303	98%	0	1	0
304	98%	1	0	0
305	98%	0	0	0
306	99%	1	0	0
307	100%	0	0	1
308	99%	0	1	0
309	100%	0	1	0
310	97%	1	0	0
311	99%	0	0	1
312	96%	0	0	1
313	98%	1	0	0
314	98%	1	0	0
315	99%	0	0	1
316	99%	0	0	1
317	99%	0	0	1
318	99%	1	0	0
319	98%	0	0	1
320	97%	0	0	1
321	99%	1	0	0
322	99%	0	0	1
323	99%	0	0	1
324	100%	1	0	0
325	99%	1	0	0
326	99%	1	0	0
327	99%	1	0	0
328	99%	1	0	0
329	99%	1	0	0
330	96%	1	0	0

ID	Percent_identity	Num_deleletion	Num_insertion	Num_pre_stop
331	98%	0	0	1
332	99%	0	0	1
333	99%	0	0	1
334	99%	0	0	1
335	99%	0	0	1
336	99%	0	1	0
337	99%	1	0	0
338	98%	0	1	0
339	98%	1	0	0
340	99%	0	0	1
341	99%	1	0	0
342	99%	1	0	0
343	99%	1	0	0
344	97%	0	0	1
345	99%	0	0	1
346	99%	1	0	0
347	99%	0	0	1
348	98%	0	1	0
349	98%	0	1	0
350	99%	1	0	0
351	98%	1	0	0
352	98%	0	0	2
353	99%	0	1	1
354	99%	1	0	1
355	99%	2	0	0
356	99%	1	0	1
357	98%	1	0	1
358	99%	1	0	1
359	99%	1	0	1
360	99%	2	1	0

ID	Percent_identity	Num_deleletion	Num_insertion	Num_pre_stop
361	99%	2	0	1
362	99%	0	1	3
363	97%	3	0	1
364	77%	7	1	1
365	87%	12	6	0
366	99%	0	1	0
367	99%	1	0	0
368	99%	1	0	0
369	99%	1	0	0
370	99%	0	0	1
371	98%	0	0	1
372	90%	0	1	0
373	99%	0	1	0
374	100%	1	0	0
375	99%	1	0	0
376	100%	1	0	0
377	100%	1	0	0
378	99%	1	0	0

ID	Deletion_list
1	
2	-1@305;
3	-4@452;
4	
5	
6	
7	
8	-1@559;
9	
10	
11	
12	
13	
14	-9@1332;
15	
16	
17	
18	
19	
20	
21	-1@554;
22	
23	
24	-2557@-747
25	
26	
27	
28	
29	
30	

ID	Deletion_list
31	
32	
33	
34	-37@895;
35	
36	
37	-1@636;
38	
39	-2@257;
40	-1@801;
41	-1@102;
42	
43	-1@397;
44	
45	-9@5091;
46	-1@501;
47	
48	
49	
50	
51	
52	
53	
54	
55	
56	
57	
58	
59	
60	-61@828;

ID	Deletion_list
61	-1@495;
62	
63	
64	-1@33;
65	-28@471;
66	-1@216;
67	-1@380;
68	
69	-1@623;
70	-172@202;
71	
72	-1@680;
73	-1@404;
74	-1@119;
75	
76	-441@-189
77	
78	
79	
80	-1@642;
81	-1@984;
82	-1@1970;
83	-11@322;
84	-1@218;
85	-1@109;
86	
87	-32@89;
88	-11@680;
89	-10@438;-12@653;
90	-1@145;-1@426;

ID	Deletion_list
91	-1@736;-1@1095;-1@1212;
92	-126@-6;-75@129;-5@210;-221@227;
93	-121@-59;-74@74;-32@155;-3@200;-190@224;-88@419;-102@515;
94	
95	-1@491;
96	
97	
98	
99	
100	-1@807;
101	
102	
103	
104	-148@113;
105	
106	
107	-1@235;
108	-4@199;
109	-657@66;
110	-1@834;
111	-11@562;
112	
113	
114	
115	
116	
117	-1@691;
118	
119	
120	

ID	Deletion_list
121	-11@182;
122	
123	
124	-29@1286;
125	
126	
127	-6@270;
128	-258@151;
129	-2@267;
130	
131	
132	-1@275;
133	
134	
135	
136	
137	-1@210;
138	
139	-4@71;
140	-356@-110
141	-1@316;
142	-5@545;
143	-1@175;
144	-2@1153;
145	-122@713;
146	
147	
148	
149	-1@188;
150	

ID	Deletion_list
151	-1@105;
152	
153	
154	
155	-253@124;
156	
157	
158	
159	
160	-8@749;
161	-573@42;
162	
163	-1@933;
164	
165	-1@358;
166	-11@53;
167	-23@270;
168	
169	
170	-1@495;
171	-1@318;
172	-17@727;
173	
174	-37@337;
175	-1@736;
176	
177	-2@40;
178	
179	-139@780;
180	-2@428;

ID	Deletion_list
181	
182	
183	
184	-1@235;
185	-1@806;
186	
187	-2@5117;
188	-6@4759;
189	
190	
191	-1@816;
192	-1@1961;
193	
194	
195	-1@597;
196	-7@726;
197	
198	-1@471;
199	
200	
201	
202	-4@1269;
203	
204	
205	
206	
207	-1@552;
208	-1@1390;
209	-1@1263;
210	-1@121;

ID	Deletion_list
211	
212	-1@632;
213	-1@817;
214	-11@562;
215	
216	
217	-47@657;
218	-657@66;
219	
220	
221	-1@759;
222	-1@1452;
223	-8@512;
224	-1@673;
225	-1@373;
226	
227	-1@625;
228	-16@1076;
229	
230	
231	-1@224;
232	-6@169;-2@844;
233	-11@197;
234	
235	
236	-4@167;
237	-1@884;
238	-52@1119;
239	
240	-1@477;

ID	Deletion_list
241	-1@453;-6@1320;
242	
243	-4@839;
244	
245	-1@123;
246	-1@225;
247	-20@764;
248	
249	
250	
251	
252	-7@139;
253	-38@116;
254	
255	
256	-4@186;
257	
258	-66@792;
259	-1@4;-18@140;
260	-25@163;
261	
262	
263	
264	-1@527;
265	
266	
267	-1@790;
268	-1@79;
269	
270	

ID	Deletion_list
271	
272	-1@648;
273	
274	-6@327;
275	-1@148;
276	
277	
278	-1@316;
279	-1@289;
280	
281	
282	
283	-1@481;
284	
285	-136@70;
286	
287	-1260@-409
288	
289	-5@1397;
290	-8@1034;
291	-205@396;
292	-1@326;
293	
294	
295	-1@68;
296	
297	-1@275;
298	
299	-1@169;
300	-153@320;

ID	Deletion_list
301	-1@64;
302	-1@348;
303	
304	-1@41;
305	
306	-2@428;
307	
308	
309	
310	-1@278;
311	
312	
313	-1@1149;
314	-1@259;
315	
316	
317	
318	-1@428;
319	
320	-6@367;
321	-11@1124;
322	
323	
324	-1@234;
325	-1@1069;
326	-1@779;
327	-10@282;
328	-1@1505;
329	-1@374;
330	-1@568;

ID	Deletion_list
331	
332	
333	
334	
335	
336	
337	-1@340;
338	
339	-1@327;
340	
341	-1@191;
342	-1@873;
343	-2@445;
344	
345	
346	-80@262;
347	
348	
349	
350	-180@216;
351	-1@225;
352	
353	
354	-1@24;
355	-1@551;-1@883;
356	-1@772;
357	-1@130;
358	-1@386;
359	-1@162;
360	-149@398;-1@741;

ID	Deletion_list
361	-1@229;-1@518;
362	
363	-1@222;-1@814;-4@1211;
364	-121@-59;-74@74;-32@155;-3@200;-190@224;-88@419;-102@515;
365	-9@113;-2@236;-1@246;-6@372;-1@494;-3@509;-6@716;-3@732;-756@2335;-372@4348;-9@5027;-9@5095;
366	
367	-7@883;
368	-1@795;
369	-1@3139;
370	
371	
372	
373	
374	-1@557;
375	-1@236;
376	-1@463;
377	-657@66;
378	-1@538;

ID	Insertion_list	Pre_stop_list	Num_inactivation
1		*1638@2622;	1
2			1
3			1
4	+1@533;		1
5		*741@3168;	1
6	+2@1560;		1
7		*234@654;	1
8			1
9	+8@872;		1
10	+1@184;		1
11	+1@169;		1
12	+1@425;		1
13		*240@573;	1
14		*132@1473;	1
15		*411@1140;	1
16	+1@873;	*813@984;	1
17	+1@699;		1
18		*339@723;	1
19	+2@1758;		1
20		*816@1908;	1
21			1
22	+1338@420;		1
23	+1@334;		1
24			1
25		*765@1041;	1
26		*774@1254;	1
27		*480@873;	1
28		*588@3048;	1
29		*2607@3741;	1
30		*471@666;	1

ID	Insertion_list	Pre_stop_list	Num_inactivation
31	+1@814;		1
32		*1059@1509;	1
33		*273@558;	1
34			1
35	+1@328;		1
36	+1@1230;		1
37			1
38		*132@912;	1
39			1
40			1
41			1
42		*366@495;	1
43			1
44		*1065@1701;	1
45	+1@2967;		1
46			1
47		*258@1221;	1
48		*273@1341;	1
49	+1338@340;		1
50		*1143@1473;	1
51		*195@987;	1
52	+1@170;		1
53		*639@2100;	1
54	+2@76;		1
55		*963@1716;	1
56		*228@1110;	1
57		*528@696;	1
58		*1437@1956;	1
59		*783@1311;	1
60			1

ID	Insertion_list	Pre_stop_list	Num_inactivation
61			1
62		*396@1329;	1
63		*744@1041;	1
64			1
65			1
66			1
67			1
68		*519@1452;	1
69			1
70			1
71		*588@3051;	1
72			1
73			1
74			1
75		*33@345;	1
76			1
77		*420@2148;	1
78		*69@267;	1
79	+5@237;		1
80			1
81			1
82		*402@2100;	2
83	+8@741;		2
84	+1@263;		2
85	+1@474;		2
86		*273@1887;*780@1887;	2
87		*396@540;	2
88	+12@360;		2
89			2
90			2

ID	Insertion_list	Pre_stop_list	Num_inactivation
91			3
92			4
93		*513@576;	8
94	+1@977;		1
95			1
96		*618@1494;	1
97		*516@1239;	1
98		*540@723;	1
99		*417@1242;	1
100			1
101	+1@608;		1
102		*792@1290;	1
103		*5235@7308;	1
104			1
105	+1@450;		1
106		*570@954;	1
107			1
108			1
109			1
110			1
111			1
112		*48@705;	1
113		*93@2430;	1
114	+1@151;		1
115		*213@714;	1
116		*759@2997;	1
117			1
118		*510@1554;	1
119		*198@2391;	1
120		*1611@2547;	1

ID	Insertion_list	Pre_stop_list	Num_inactivation
121			1
122		*51@477;	1
123	+3@124;	*177@840;	1
124			1
125		*372@1053;	1
126		*2229@2562;	1
127	+1@1305;		1
128			1
129			1
130		*867@1773;	1
131		*423@855;	1
132			1
133		*1302@1665;	1
134		*783@1095;	1
135		*945@1929;	1
136		*552@2145;	1
137			1
138		*9@1128;	1
139			1
140			1
141			1
142			1
143			1
144			1
145			1
146		*288@459;	1
147		*219@849;	1
148	+4@832;		1
149			1
150	+1@2157;		1

ID	Insertion_list	Pre_stop_list	Num_inactivation
151			1
152	+1@1543;		1
153	+1@499;		1
154		*978@1380;	1
155			1
156		*453@1254;	1
157		*540@897;	1
158		*123@738;	1
159		*384@1002;	1
160			1
161			1
162		*201@1344;	1
163			1
164	+1@882;		1
165			1
166			1
167			1
168		*375@1662;	1
169		*327@1098;	1
170			1
171			1
172			1
173	+8@239;		1
174			1
175			1
176	+1@819;		1
177			1
178	+1@463;		1
179			1
180			1

ID	Insertion_list	Pre_stop_list	Num_inactivation
181		*711@1557;	1
182	+1@122;		1
183		*747@1422;	1
184			1
185			1
186		*171@576;	1
187			1
188	+1@3200;		1
189	+1@1644;		1
190	+1@565;		1
191			1
192			1
193	+1@958;		1
194		*447@1053;	1
195			1
196			1
197		*60@402;	1
198		*1488@1539;	1
199		*168@2664;	1
200		*168@762;	1
201		*732@1008;	1
202			1
203	+8@345;		1
204		*504@867;	1
205		*456@999;	1
206		*351@1245;	1
207			1
208			1
209			1
210			1

ID	Insertion_list	Pre_stop_list	Num_inactivation
211		*324@690;	1
212			1
213			1
214			1
215		*552@903;	1
216		*219@879;	1
217			1
218			1
219		*477@1506;	1
220		*99@993;	1
221			1
222			1
223			1
224			1
225			1
226	+1@1459;		1
227			1
228			1
229		*684@996;	1
230		*24@486;	1
231			1
232			1
233			1
234		*540@753;	1
235		*510@2061;	1
236			1
237			1
238			1
239	+1@1055;		1
240			1

ID	Insertion_list	Pre_stop_list	Num_inactivation
241			1
242		*93@2430;	1
243			1
244		*192@858;	1
245			1
246			1
247			1
248		*216@1164;	1
249		*228@741;	1
250		*48@447;	1
251		*318@1221;	1
252			1
253			1
254	+1@161;		1
255		*186@684;	1
256	+1@842;		2
257		*105@765;*441@765;	2
258		*1341@2349;	2
259			2
260		*252@972;*456@972;	3
261	+18@334;+23@1185;	*204@2982;	4
262	+2@413;		1
263	+1@128;		1
264			1
265		*606@1173;	1
266		*66@516;	1
267			1
268			1
269		*429@1455;	1
270		*141@534;	1

ID	Insertion_list	Pre_stop_list	Num_inactivation
271		*237@1137;	1
272			1
273	+1@291;		1
274	+1@98;		1
275			1
276		*723@1233;	1
277	+1@710;		1
278			1
279			1
280		*141@573;	1
281	+1@989;		1
282		*255@459;	1
283			1
284	+1@136;		1
285			1
286		*534@1803;	1
287			1
288	+1@163;		1
289			1
290			1
291			1
292			1
293		*150@885;	1
294		*276@1713;	1
295			1
296		*1170@1872;	1
297			1
298		*255@912;	1
299			1
300			1

ID	Insertion_list	Pre_stop_list	Num_inactivation
301			1
302			1
303	+8@399;		1
304			1
305			1
306			1
307		*894@1323;	1
308	+1@348;		1
309	+1@1056;		1
310			1
311		*384@1188;	1
312		*252@2193;	1
313			1
314			1
315		*72@1014;	1
316		*96@450;	1
317		*408@1335;	1
318			1
319		*264@588;	1
320		*330@867;	1
321			1
322		*1296@1644;	1
323		*525@828;	1
324			1
325		*1794@2019;	1
326			1
327			1
328			1
329			1
330			1

ID	Insertion_list	Pre_stop_list	Num_inactivation
331		*237@1242;	1
332		*483@1428;	1
333		*75@348;	1
334		*351@834;	1
335		*204@861;	1
336	+1@206;		1
337			1
338	+2@670;		1
339			1
340		*399@1311;	1
341			1
342			1
343			1
344		*435@1098;	1
345		*462@2088;	1
346			1
347		*396@627;	1
348	+1@53;		1
349	+1@1209;		1
350			1
351			1
352		*921@2622;*1953@2622;	2
353	+1@256;	*285@450;	2
354		*507@1374;	2
355			2
356		*177@1332;	2
357		*147@360;	2
358		*201@789;	2
359		*63@2538;	2
360	+20@867;		3

ID	Insertion_list	Pre_stop_list	Num_inactivation
361		*504@1281;	3
362	+1@3247;	*1362@3543;*1785@3543;*3426@3543;	3
363		*159@2349;	4
364	+2@219;	*513@576;	9
365	+6@312;+3@400;+4@488;+6@615;+12@908;+4@5184;		18
366	+4@451;		1
367			1
368			1
369			1
370		*978@1380;	1
371		*102@366;	1
372	+1@128;		1
373	+1@778;		1
374			1
375			1
376			1
377			1
378			1

ID	Note
1	
2	
3	
4	
5	
6	
7	
8	
9	
10	
11	
12	
13	
14	ignore the 9-bp deletion
15	
16	ignore the pre-mature stop
17	
18	
19	
20	
21	
22	insertion is a transposase of Tn10
23	
24	indel size was manually corrected
25	
26	
27	
28	
29	
30	

ID	Note
31	
32	
33	
34	
35	
36	
37	
38	
39	
40	
41	
42	
43	
44	
45	ignore the 9-bp deletion
46	
47	
48	
49	insertion is a transposase of Tn10
50	
51	
52	
53	
54	
55	
56	
57	
58	
59	
60	

ID	Note
61	
62	
63	
64	
65	
66	
67	
68	
69	
70	
71	
72	
73	
74	
75	
76	indel size was manually corrected
77	
78	
79	
80	
81	
82	
83	
84	
85	
86	
87	
88	
89	
90	

ID	Note
91	
92	indel size was manually corrected
93	possible mis-alignment, indel size was manually corrected
94	
95	
96	
97	
98	
99	
100	
101	
102	
103	
104	
105	
106	
107	
108	
109	
110	
111	
112	
113	
114	
115	
116	
117	
118	
119	
120	

ID	Note
121	
122	
123	ignore the 3-bp insertion
124	
125	
126	start: ATG->ATT, ignore the pre-mature stop
127	ignore the 6-bp deletion
128	
129	
130	
131	
132	
133	
134	
135	
136	
137	
138	
139	
140	indel size was manually corrected
141	
142	
143	
144	
145	
146	
147	
148	
149	
150	

ID	Note
151	
152	
153	
154	
155	
156	
157	
158	
159	
160	
161	
162	
163	
164	
165	
166	
167	
168	
169	
170	
171	
172	
173	
174	
175	
176	
177	
178	
179	indel size was manually corrected
180	

ID	Note
181	
182	
183	
184	
185	
186	
187	
188	ignore the 6-bp deletion
189	
190	
191	
192	
193	
194	
195	
196	
197	
198	ignore the pre-mature stop
199	
200	
201	
202	
203	
204	
205	
206	
207	
208	
209	
210	

ID	Note
211	
212	
213	
214	
215	
216	
217	
218	
219	
220	
221	
222	
223	
224	
225	
226	
227	
228	
229	
230	
231	
232	ignore the 6-bp deletion
233	
234	
235	
236	
237	
238	
239	
240	

ID	Note
241	ignore the 6-bp deletion
242	
243	
244	
245	
246	
247	
248	
249	
250	
251	
252	
253	
254	
255	
256	
257	
258	
259	
260	
261	also contains a 873-bp inversion
262	
263	
264	
265	
266	
267	
268	
269	
270	

ID	Note
271	
272	
273	
274	ignore the 6-bp deletion
275	
276	
277	
278	
279	
280	
281	
282	
283	
284	
285	
286	
287	indel size was manually corrected
288	
289	
290	
291	
292	
293	
294	
295	
296	
297	
298	
299	
300	

ID	Note
301	
302	
303	
304	
305	start: ATG->ATA
306	
307	
308	
309	
310	
311	
312	
313	
314	
315	
316	
317	
318	
319	
320	ignore the 6-bp deletion
321	
322	
323	
324	
325	ignore the pre-mature stop
326	
327	
328	
329	
330	

ID	Note
331	
332	
333	
334	
335	
336	
337	
338	
339	
340	
341	
342	
343	
344	
345	
346	
347	
348	
349	
350	
351	
352	
353	
354	
355	
356	
357	
358	
359	
360	

ID	Note
361	
362	ignore 1 pre-mature stop
363	
364	possible mis-alignment, indel size was manually corrected
365	possible mis-alignment
366	
367	
368	
369	
370	
371	
372	
373	
374	
375	
376	
377	
378	
